# Patient-derived teratomas as a humanized platform for dystrophin restoration by ex vivo cell therapy and in vivo base editing

**DOI:** 10.1038/s12276-026-01854-5

**Published:** 2026-10-01

**Authors:** Mihn Jeong Park, Hyeon-Bin Kim, Yuna Hwang, Yun-Jeong Kim, Ju-Chan Park, Ho-Kyun Lim, Yun-Gwi Park, SungHwan Moon, Hyukjin Lee, Hyuk-Jin Cha

**Affiliations:** 1 College of Pharmacy, Seoul National University, Seoul, Republic of Korea; 2 College of Pharmacy, Ewha Womans University, Seoul, Republic of Korea; 3 College of Biotechnology and Natural Resources, Chung-Ang University, Anseong, Republic of Korea; 4 School of Transdisciplinary Innovations, Seoul National University, Seoul, Republic of Korea

**Keywords:** Targeted gene repair, Cell delivery

## Abstract

Cell and gene therapies hold great promise for treating monogenic disorders, yet their preclinical evaluation remains limited by the lack of scalable, human-specific models. Here we establish a patient-derived teratoma xenograft platform as a proof-of-concept system to evaluate ex vivo and in vivo therapeutic strategies for Duchenne muscular dystrophy (DMD). Teratomas generated from DMD patient-derived induced pluripotent stem cells contained mesodermal derivatives, including skeletal muscle-like tissue, enabling assessment of dystrophin restoration after therapeutic intervention. For ex vivo cell therapy, myogenic progenitors derived from adenine base editor-corrected induced pluripotent stem cells were transplanted into DMD teratomas, resulting in partial restoration of shorter dystrophin isoforms but not reproducible recovery of full-length Dp427m. For in vivo gene editing, local delivery of adenine base editor mRNA encapsulated in lipid nanoparticles induced dose-dependent editing and restoration of shorter dystrophin isoforms, including Dp71. Full-length, muscle-specific Dp427m was detected only in a subset of sequentially matured secondary teratomas with enriched muscle differentiation, indicating that myogenic maturation is a critical determinant of this therapeutic readout. Together, these findings support patient-derived DMD teratomas as an exploratory humanized platform for evaluating patient-specific gene correction and dystrophin isoform restoration, while highlighting the need for further optimization to reproducibly model full-length Dp427m recovery.

## Patient-derived teratomas advance Duchenne muscular dystrophy therapies

Duchenne muscular dystrophy (DMD) is a severe genetic disorder caused by mutations in the *DMD* gene, leading to progressive muscle degeneration. This study explores innovative therapeutic strategies using patient-derived induced pluripotent stem (iPS) cells and gene editing technologies. Researchers developed a DMD-teratoma model from patient iPS cells, which mimics the absence of dystrophin, a key protein missing in DMD. They demonstrated that both ex vivo therapy with gene-corrected iPS cell-derived myogenic progenitors and in vivo adenine base editing could restore dystrophin expression in this model. The study used advanced techniques such as lipid nanoparticle-mediated delivery of base editors to achieve precise gene correction. Results showed restoration of dystrophin isoforms, including the full-length Dp427m, in muscle-enriched teratomas. This work highlights the potential of combining cell and gene therapies to address the diverse mutations in DMD, paving the way for future personalized treatments. This summary was initially drafted using artificial intelligence, then revised and fact-checked by the author.

## Introduction

Cell and gene therapies have rapidly advanced as transformative modalities for inherited disorders that were once considered incurable^1,2^. In vivo gene therapy using adeno-associated virus (AAV) vectors has achieved durable gene delivery and functional benefit across several genetic diseases^3^, while ex vivo approaches have traditionally centered on hematopoietic stem cells owing to their robust regenerative capacity and the feasibility of autologous transplantation following gene correction or transgene integration^4^. The extension of ex vivo strategies beyond hematopoietic disorders to tissue-specific genetic diseases is exemplified by the recent US Food and Drug Association (FDA) approvals of Zevaskyn (Prademagene Zamikeracel), an autologous epidermal cell therapy delivering intact COL7A1 for recessive dystrophic epidermolysis bullosa, and Encelto (Revakinagene Taroretcel), an allogeneic retinal pigment epithelial cell therapy secreting recombinant human ciliary neurotrophic factor for idiopathic macular telangiectasia type 2 (refs. ^5,6^). The advent of safe and efficient gene editing of patient-derived induced pluripotent stem (iPS) cells not only facilitates the generation of isogenic disease models but also provides a renewable source of autologous cells for therapeutic transplantation^7,8^.

Duchenne muscular dystrophy (DMD), an X-linked disorder caused by mutations in the gene *DMD* that abolish dystrophin expression, is characterized by progressive muscle degeneration, loss of ambulation and premature mortality^9^. Although FDA-approved antisense oligonucleotide therapies targeting exon skipping are available, they achieve only limited dystrophin restoration; accordingly, cell-based strategies using patient-derived iPS cell-based myogenic progenitors have been extensively investigated as alternative therapeutic approaches^10^. Established directed differentiation protocols can generate skeletal myogenic cells from human pluripotent stem cells^11,12^, and complementary teratoma-based approaches have shown that in vivo differentiation can produce functional myogenic progenitors from mouse and human pluripotent stem cells^13,14^. Clinical-grade production of iPS cell-derived myogenic progenitors has recently been achieved, yielding the product MyoPAXon, which aims to provide an off-the-shelf cell therapy for muscular dystrophies and has successfully been used to restore dystrophin expression in dystrophic mouse and nonhuman primate models^15^. Beyond these approaches, in vivo AAV-based delivery of mini-dystrophin^16^ and precise genome-editing strategies—including exon skipping^17,18^, base editing^19,20^ and prime editing^21^—have expanded the therapeutic toolbox for correcting the wide spectrum of pathogenic variants observed in patients with DMD^22^.

Despite the promise of these therapies, their translational evaluation remains constrained. The *mdx* mouse model, which carries a nonsense mutation in the *Dmd* gene^23^, and its subsequent variants^24^ have been widely used for both mechanistic studies and translational research. However, murine *Dmd* differs substantially from its human counterpart in sequence, exon–intron organization and splicing regulation, and the phenotypes resulting from its alteration are relatively mild. Consequently, these models are inadequate for directly modeling many patient-specific mutations^24^. To address this limitation, diverse humanized DMD mouse models have been generated by replacing murine exons with human sequences, thereby allowing genome-editing tools designed for human mutations to be tested in vivo^20,25–27^. While these models provide proof of concept for editing at authentic human sites, each line corresponds to a specific mutation and thus captures only a narrow fraction of the mutational spectrum. Moreover, generating individual humanized mouse models for each clinically relevant mutation is a highly laborious and time-consuming process, limiting their scalability and translational relevance.

Here, we leveraged teratomas derived from the iPS cells of patients with DMD (DMD-teratomas), which recapitulate the absence of dystrophin expression, as a patient-derived humanized preclinical model. Using this platform, we demonstrate that both autologous ex vivo therapy with gene-corrected iPS cell-derived myogenic progenitors and in vivo adenine base editor (ABE) mRNA delivery via lipid nanoparticles (LNPs) can restore dystrophin expression within the teratoma. These findings establish DMD-teratomas as a versatile and mutation-inclusive system for evaluating and comparing cell- and gene-based therapeutic strategies for DMD.

## Material and methods

### Cell culture and iPS cell transfection/electroporation

hiPS cell lines (NM004006.2 c.433 C>T DMD iPS cell; National Institutes of Health) were maintained in iPS cell-brew MACS (Miltenyi Biotec, #130-104-368) with 0.1% gentamycin (Gibco, #15750-060) on a Matrigel (Corning, #354277)-coated cell culture dish at 37 °C and humidified to 5% in a CO_2_ incubator. On transfer, hiPS cells were rinsed with Dulbecco’s phosphate-buffered saline and detached enzymatically with Dispase (Life Technologies). Y27632 (10 μM; PeproTech #1293823) was used for 1 day after the attachment if needed. According to the standardized protocol for electroporation of human pluripotent stem cells^28^, gene delivery with the electroporator (NEPA-21, NEPAGENE), iPS cells were rinsed with DPBS and detached with Accutase (#561527, BD Bios-ciences). Cells were resuspended and diluted to 1 × 106 cells per 100 μl with Opti-MEM (#31985070, Gibco) after three rounds of Opti-MEM washing (#31985070, Gibco). A total of 3 μg of the singel guide (sg)RNA or prime editing guide (peg)RNA vector and 2 μg of the ABE vectors were added to the cell solution. The electroporation was performed with NEPA-21 electroporator with 175 V and 2.5 ms of poring pulse as described. Dissociated cells after electroporation were plated into a culture dish with 10 μM of Y27632.

### Teratoma formation

Human iPS (hiPS) cells were collected at ~70–80% confluency using Dispase (Life Technologies), washed and resuspended in iPS cell-brew MACS. Approximately 1 × 10^6^ iPS cells were injected into the testis of 4-week-old Balb-c/nu mice under isoflurane anesthesia. For experiments involving gene-edited cells, both mutant iPS (M-iPS) cells and edited iPS (E-iPS) cells were transplanted in parallel. Mice were monitored weekly for tumor growth. After 6–12 weeks, teratomas were excised, fixed in 4% paraformaldehyde, dehydrated, embedded in paraffin and sectioned at 7 μm. Hematoxylin and eosin staining was performed to confirm the presence of derivatives of all three germ layers. For molecular analyses, fresh teratoma tissue was snap-frozen in liquid nitrogen for protein or nucleic acid extraction. All animal procedures were approved by the Institutional Animal Care and Use Committee (IACUC) of Seoul National University (institutional review board (IRB) no. 2310/001-027) and were performed in accordance with institutional and national guidelines for animal welfare.

### CM differentiation

DMD-iPS cells were maintained on matrigel-coated plates in iPS cell-brew MACS medium and differentiated into CMs following a chemically defined monolayer protocol. In brief, cells at ~90% confluence were treated with 2–3 µM CHIR99021 (Biogems) for 48 h to activate Wnt signaling, followed by 1–2 µM Wnt C59 (Selleckchem) for 48 h to promote cardiac specification. The culture medium (Roswell Park Memorial Institute medium (RPMI) 1640 supplemented with B27 minus insulin) was subsequently replaced every 2 days until spontaneous contractile CMs appeared at day 10–12. Beating clusters were metabolically purified using lactate-based selection, and the resulting CMs were maintained in RPMI 1640/B27 (Gibco) medium for downstream analyses.

### Myotube differentiation

Both M-iPS and E-iPS cells were dissociated into single cells and seeded on Matrigel-coated plates, followed by induction of paraxial mesoderm using 3 µM CHIR99021 and 0.5 µM LDN193189 (Stemgent) in serum-free DMEM/F12 medium supplemented with ITS and FGF2 (Peprotech). Cultures were maintained for 30–40 days with sequential media changes to promote myogenic commitment and fusion. After subculturing in Skeletal Muscle Growth Medium (Lonza), secondary differentiation was induced by switching to N2- or horse serum-based medium up to day 90, leading to the formation of multinucleated, striated myotubes expressing MyHC, MYOG and dystrophin. The resulting cultures contained both mature myofibers and Pax7^+^ satellite-like progenitors suitable for downstream analysis.

### Myo-progenitor engraftment

Using the above differentiation protocol, E-iPS cells were differentiated up to day 12 to obtain myo-progenitor-stage cells. The resulting myo-progenitors were dissociated with TrypLE Express, and approximately 1 × 10^5^ cells were injected into M-teratomas using a 23-gauge needle. Following transplantation, teratoma-bearing mice were maintained for ~4 weeks to allow engraftment and proliferation of the transplanted cells. Subsequently, teratomas were surgically excised and collected for downstream analyses.

### Teratoma transplantation

Teratomas were excised and washed thoroughly in sterile PBS, then cut into approximately 1-cm^3^ pieces. Each fragment was transplanted subcutaneously into BALB/c nude (OrientBio) mice under sterile conditions. Following implantation, the surgical sites were closed appropriately to ensure recovery and prevent infection.

### IVT mRNA synthesis

DNA templates for in vitro transcription (IVT) were generated from the plasmid pCMV-SpRY-ABE8e (Addgene, #185671) by PCR amplification using PrimeSTAR HS DNA Polymerase (Takara), followed by linearization. A 120-nucleotide poly A-tail was encoded in the template using a primer containing a poly T sequence during PCR. The PCR products were purified with the AccuPrep PCR/Gel Purification Kit (Bioneer) and verified by electrophoresis on a 1% agarose gel in 1× TBE buffer. smRNA was synthesized using the MEGAscript T7 Transcription Kit (Thermo Fisher Scientific), with CleanCap AG (TriLink) included in the reaction and UTP substituted with *N*^1^-methyl-pseudouridine-5′-triphosphate (N^1^-Me-ΨUTP). Transcription reactions were carried out at 37 °C for 2 h, followed by DNase treatment. The resulting mRNA was purified using the HiGene DNA/RNA Purification Mini Column (Biofact). Transcript integrity was confirmed by electrophoresis on a 1% denaturing agarose gel in 1× 3-(*N*-morpholino)propanesulfonic acid (MOPS) buffer.

### Preparation and characterization of LNPs

LNPs were prepared using a microfluidic mixing system (Ignite, Precision NanoSystems). A lipid mixture consisting of a proprietary ionizable lipid (undisclosed structure), 1,2-distearoyl-*sn*-glycero-3-phosphocholine (DSPC), cholesterol and a polyethylene glycol (PEG)-lipid was dissolved in ethanol according to a defined molar ratio. The ethanol phase was prepared by mixing lipids in ethanol (97.5% v/v) with 10 mM citrate buffer, pH 3.0 (2.5% v/v). ABE mRNA and sgRNA were diluted in an aqueous phase composed of 1× PBS and 10 mM citrate buffer (v/v of 2:1). LNPs were formulated by microfluidic mixing at a total flow rate of 12 ml min^−1^, with an ethanol-to-aqueous phase ratio of 1:3. The resulting LNPs were diluted in 40 volumes of 1× PBS and concentrated by ultrafiltration using Amicon Ultra-15 centrifugal filters, 10 kDa MWCO (Millipore). The mean particle size and polydispersity index (PDI) of LNPs were determined using a Zetasizer Lab Blue (Malvern Instruments) following dilution to 0.5 μg ml^−1^ in PBS. RNA encapsulation efficiency was determined with the Quant-iT RiboGreen Assay (Thermo Fisher Scientific), and fluorescence was measured using a SpectraMax iD3 microplate reader (Molecular Devices).

### NGS sequencing

As previously described^29^, genomic DNA was isolated from edited and control samples, and target as well as predicted off-target loci were amplified by PCR using locus-specific primers. Amplicon libraries were subjected to paired-end sequencing (2 × 150 base pairs (bp)) on an Illumina MiniSeq platform. Sequencing data were analyzed by alignment to the reference amplicon sequence, and editing frequencies were determined from the proportion of variant reads at the target nucleotide position. Only variants passing predefined thresholds for read depth, supporting read count and variant allele frequency were included in downstream analyses. Background variants detected in untreated controls were excluded. Editing outcomes were evaluated primarily within the expected ABE editing window, while variants located in primer regions, read ends, repetitive sequences or homopolymeric regions were excluded as potential technical artifacts.

### scRNA sequencing

Single-cell transcriptomic profiling was performed using the 10x Genomics Chromium GEM-X Single Cell 3′ RNA-seq workflow. In brief, dissociated single-cell suspensions were loaded onto the Chromium controller to generate gel beads-in-emulsion (GEMs), in which individual cells were co-encapsulated with barcoded gel beads for cell-specific 10x barcodes and unique molecular identifiers (UMIs). Reverse transcription and cDNA amplification were carried out according to the Chromium Single Cell 3′ v4 (GEM-X) protocol to generate 3′ gene expression libraries, which were subsequently sequenced on an Illumina platform using paired-end reads (read 1: 28-bp cell barcode/UMI; read 2: 90-bp transcript insert; dual 10-bp i7/i5 sample indexes). Base call files (BCL) were converted to FASTQ with BCL Convert, and libraries were processed using Cell Ranger (v9.0.1) for alignment to the GRCh38 reference (2024-A), UMI counting, cell barcode filtering and generation of gene-by-cell count matrices. Cell Ranger ‘count’ was further used to perform dimensionality reduction (principal component analysis), clustering and gene expression quantification for downstream analyses.

### Primary data processing

Raw sequencing BCL files were demultiplexed into FASTQ files using the cellranger mkfastq pipeline. The resulting FASTQ files were processed with Cell Ranger (10x Genomics, v9.0.1) count to align reads to a GRCh38 reference genome and generate a feature-barcode count matrix. This matrix, containing UMI counts per gene for each cell, served as the input for all subsequent analyses. All processing was performed using a consistent version of the Cell Ranger software to ensure reproducibility.

### Single-cell RNA data analysis

Single-cell RNA sequencing (RNA-seq) datasets from samples were processed using Suerat v5.2.1. Cells with fewer than 1000 UMIs, fewer than 1000 detected genes, more than 20% mitochondrial transcripts or more than 10% ribosomal protein-coding transcripts were removed. Normalization and variance stabilization were performed using an SCTransform-based approach, and the datasets were integrated after selecting 3000 highly variable features. Batch effects were corrected during the integration step. The integrated data were dimensionally reduced with principal component with principal component analysis, and the first 18 principal components were used for constructing a shared nearest-neighbor graph and subsequent clustering. Low-dimensional visualization was performed with UMAP to explore cellular heterogeneity. Cell clusters were annotated into 20 cell types representing the three germ layers on the basis of the marker genes and their match to canonical markers.

### Statistical analysis

The graphical and quantitative data were presented as mean ± s.d. Statistical significance among the three groups and between groups was determined using one-way or two-way analysis of variance following Tukey’s post hoc test and Student’s *t-*test, respectively. Statistical analysis was performed with GraphPad Prism 10 software (https://www.graphpad.com/scientific-software/prism/). Significance was assumed for *P* < 0.05 (*), *P* < 0.01 (**), *P* < 0.001 (***) and *P* < 0.0001 (****).

## Results

### Establishment of a DMD iPS cell isogenic pair with base editor

**Fig. 1:**
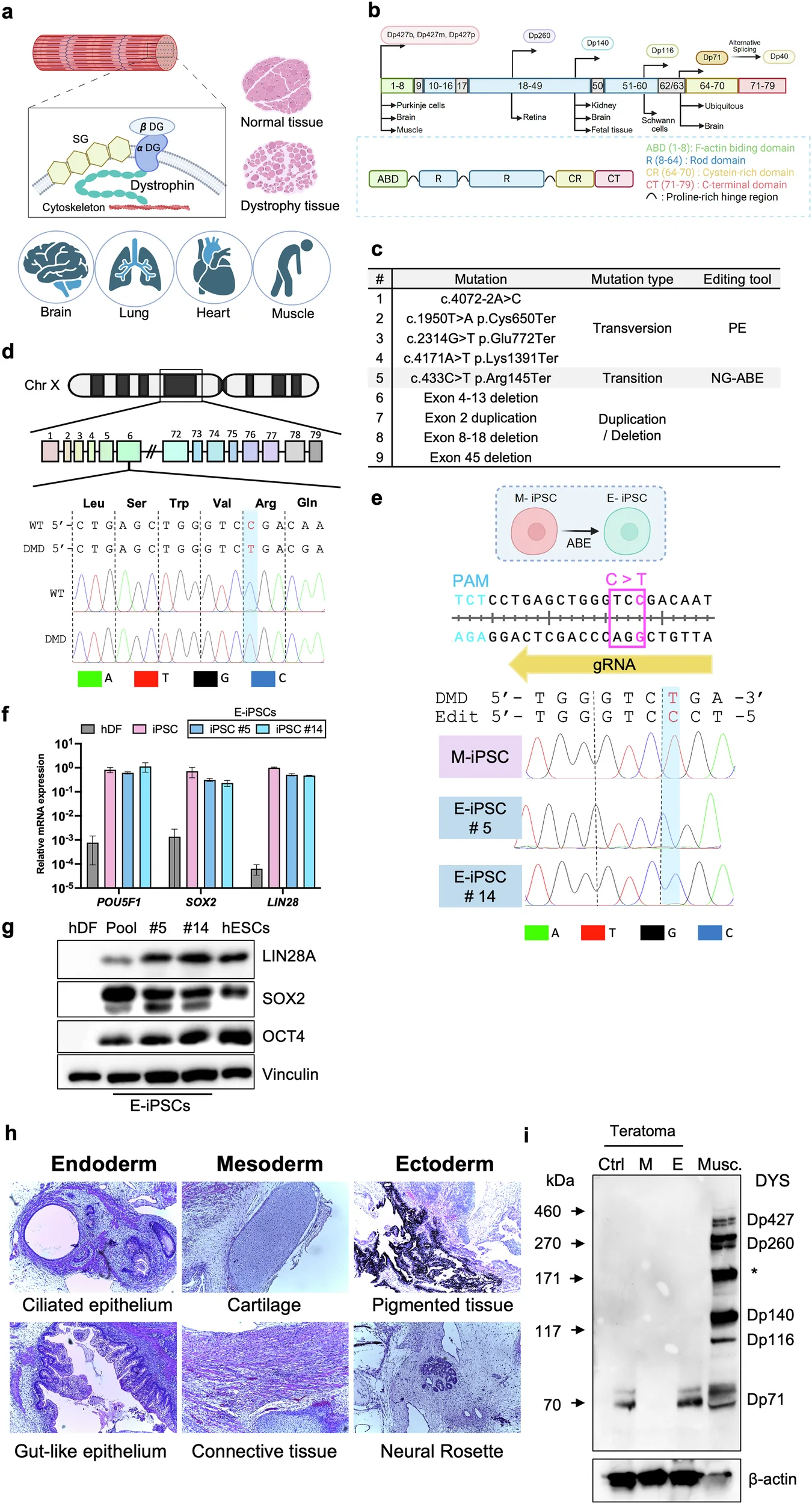
Establishment of isogenic DMD iPS cell pair by base editing. **a** Diagram showing isoform-specific expression patterns of Dp427m (muscle), Dp427c/p (brain), Dp260 (retina), Dp140 (CNS/kidney), Dp116 (Schwann cells) and Dp71/Dp40 (ubiquitous). **b** Schematic representation of DMD gene structure (79 exons) and multiple dystrophin isoforms generated by alternative promoters and splicing. **c** Summary of nine DMD patient-derived iPS cell lines obtained from the Korean National Stem Cell Bank, categorized by mutation type and exon location. **d** Sanger sequencing of DMD-iPS cells (M-iPS cells) confirming the nonsense mutation (c.433 C>T; p.Arg145Ter) in exon 6. **e** ABE-mediated correction of the exon 6 mutation to generate E-iPS cells. **f** Representative reverse transcription quantitative PCR (RT–qPCR) showing comparable expression of pluripotency markers (POU5F1, SOX2, LIN28A) in E-iPS cells and control human embryonic stem cells (*n* = 3). **g** Immunoblot of pluripotency proteins OCT4, SOX2 and LIN28A in human dermal fibroblasts (hDFs), human embryonic stem cells and E-iPS cells. Vinculin served as a loading control. **h** Teratoma formation assay confirming three-germ-layer differentiation potential of E-iPS cells, showing representative histology of endodermal, mesodermal and ectodermal derivatives. **i** Immunoblot of dystrophin from teratomas derived from control (Ctrl), mutant (M) and edited (E) iPS cells compared with mouse muscle (Musc.), revealing multiple dystrophin isoforms (Dp427, Dp260, Dp140, Dp116 and Dp71). Scale bar, 50 μm.

The *DMD* gene, comprising 79 exons, encodes multiple dystrophin isoforms of varying sizes through alternative promoters and splicing; as such, mutations in different regions differentially affect isoform expression, resulting in tissue-specific phenotypes in muscle, heart, lung and brain (Fig. 1a). Dp427m, the full-length isoform, is predominantly expressed in skeletal and cardiac muscle. Additional isoforms contribute to the diverse physiological roles of dystrophin in nonmuscle tissues, including Dp427b and Dp427p in the brain, Dp260 in the retina, Dp140 in the central nervous system and kidney, Dp116 in peripheral Schwann cells and the ubiquitously expressed Dp71 and Dp40^9^ (Fig. 1b). Thus, loss of these isoforms underlies the multi-organ pathophysiology of DMD, manifesting as progressive muscle degeneration and cardiomyopathy, along with cognitive impairment and retinal dysfunction in subsets of patients. Importantly, mutation-specific DMD phenotypes arise from the depletion of distinct dystrophin isoforms depending on the location of the mutation within the *DMD* gene. Proximal mutations typically abolish all isoforms and lead to severe muscle, cardiac and cognitive manifestations, whereas distal mutations may spare some shorter isoforms, correlating with milder or tissue-restricted phenotypes.

We obtained nine DMD patient-derived iPS (DMD-iPS) cell lines with distinct mutation profiles (one missense, four nonsense, one duplication and three deletion mutations) from the Korean National Stem Cell Bank (Supplementary Fig. 1a). Based on their mutation profiles, all nine lines lack full-length dystrophin (Fig. 1c), suggesting that myopathy is a common pathogenic phenotype (Supplementary Fig. 1b). Among the nine lines, we focused on mutant 5, which carries a nonsense mutation caused by a C-to-T transition at exon 6 (Fig. 1c). This mutation is directly targetable by ABE, one of the most well-characterized gene editing tools with minimal off-target activity^7^, which has also advanced into multiple preclinical and early clinical studies in reflection of its therapeutic potential^30–32^.

Sequencing of DMD-iPS cells from mutant 5 confirmed the C-to-T nonsense mutation in exon 6 of *DMD* on the X chromosome (Fig. 1d). To restore dystrophin expression, we corrected this mutation using ABE, generating an isogenic pair of mutant and edited DMD-iPS cells (M-iPS and E-iPS cells). Two single-cell-derived edited clones were isolated and expanded (Fig. 1e). These E-iPS cells retained pluripotency, as confirmed by pluripotency gene expression, immunostaining (Fig. 1f, g), alkaline phosphatase activity (Supplementary Fig. 1c) and teratoma formation with derivatives of all three germ layers (Fig. 1h). Teratomas derived from M-iPS cells lacked dystrophin expression, whereas those from control-iPS cells (Ctrl), E-iPS cells and mouse muscle all showed robust dystrophin with multiple immunoblot bands (Fig. 1i). The presence of multiple bands with the C-terminal antibody is consistent with previous reports that, in addition to the full-length Dp427, dystrophin immunoblots frequently reveal shorter isoforms (Dp140, Dp116, Dp71) and proteolytic fragments^33,34^ (Supplementary Fig. 1d). Notably, the predominant ~71-kDa band observed in Ctrl- and E-iPS cell-derived teratomas, but absent in M-iPS cells, probably represents the ubiquitous Dp71 isoform, while other tissue-specific isoforms would be expected to emerge in more mature or lineage-restricted derivatives (Fig. 1i).

### Recovery of dystrophin expression in myocytes from DMD-iPS cell by gene editing

**Fig. 2:**
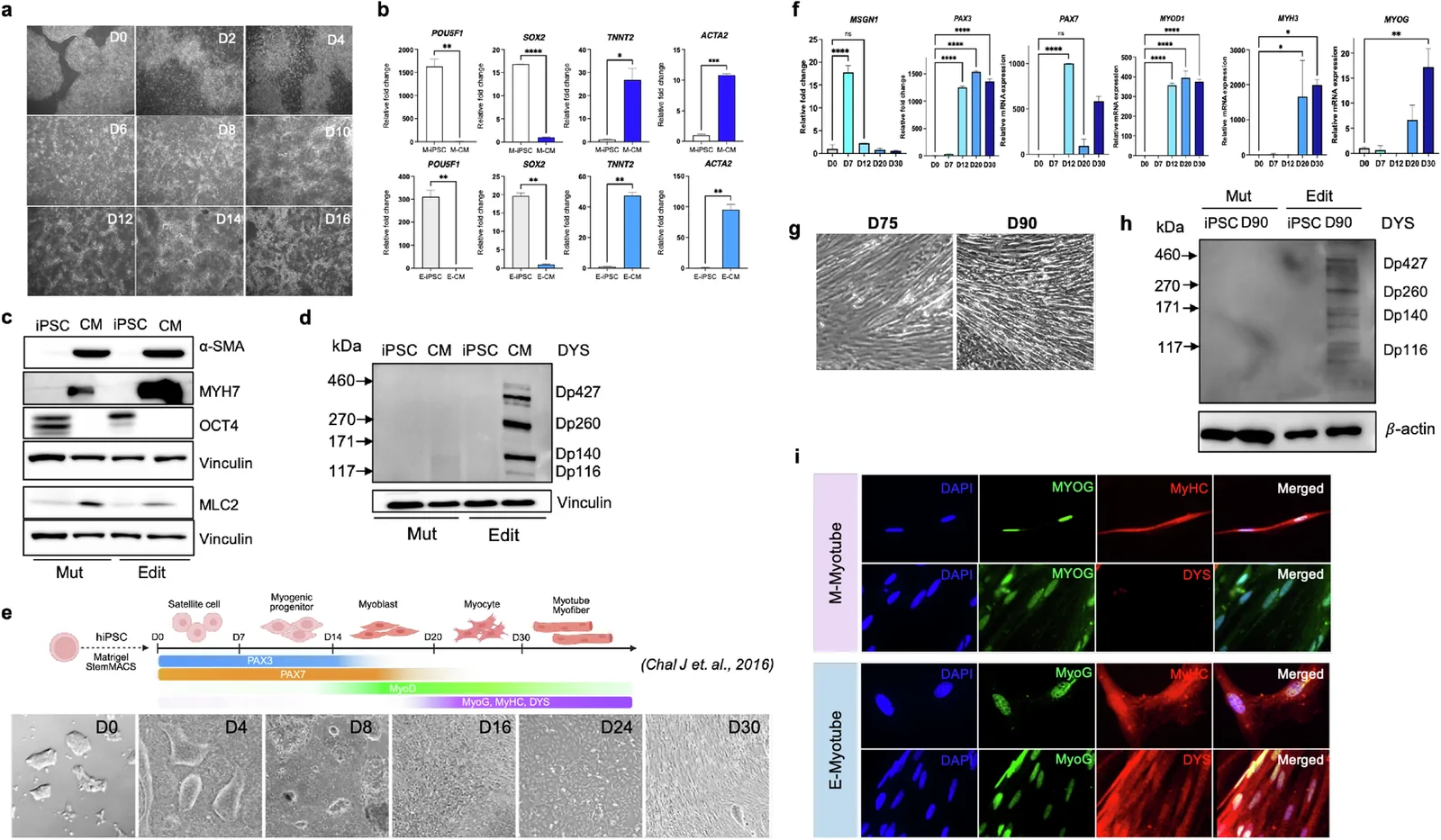
Restoration of dystrophin expression in CMs and myotubes derived from DMD-iPS cells after gene correction. **a** Bright-field images of CM differentiation from M-iPS and E-iPS cells over 16 days. **b** Relative mRNA expression of pluripotency (POU5F1, SOX2) and cardiac lineage markers (ACTA2, TNNT2) in M-CMs and E-CMs (*n* = 3; mean ± s.d.). **c** Immunoblot of OCT4, α-SMA, MYH7 and MLC2 showing comparable CM differentiation efficiency in M-iPS and E-iPS cells. **d** Immunoblot analysis of DYS protein expression in E-CM and M-CM. **e** Time-course bright-field images showing morphological transitions from iPS cells to mesodermal progenitors (D0–D30) during myogenic differentiation. **f** Representative RT–qPCR showing comparable expression of myogenic differentiation markers (MSGN1, PAX3, PAX7, MYOD1, MYH3 and MYOG). **g** Representative images of myotube formation at days 75 and 90. **h** Immunoblot of dystrophin and β-actin in mutant (M) and edited (E) myotubes showing restoration of full-length Dp427m in E-myotubes. **i** Immunofluorescence of MYOG, MyHC and DYS in M- and E-myotubes confirming dystrophin restoration on base editing. Scale bar, 50 μm.

Correction of the exon 6 nonsense mutation in DMD-iPS cells (c.433T>C) was probably to restore only the ubiquitous Dp71 isoform in teratomas (Fig. 1i). To determine whether gene editing rescued the muscle-specific full-length isoform Dp427m (~427 kDa), we differentiated the isogenic iPS cell pair into CMs, wherein Dp427m expression can be directly evaluated. Over a 16-day differentiation period, cell morphology transitioned from compact iPS cell colonies to elongated, striated and spontaneously contracting CMs (Fig. 2a and Supplementary Movie 1). Real-time PCR showed comparable downregulation of pluripotency markers (*SOX2*, *POU5F1*) and upregulation of cardiac lineage markers (*ACTA2, TNNT2*) in both M-iPS and E-iPS cells, indicating equivalent differentiation efficiency (Fig. 2b). Protein analysis likewise confirmed comparable expression of α-SMA, myosin heavy chain (MYH7) and ventricular myosin light chain 2 (MLC2), along with loss of OCT4 (Fig. 2c). Immunoblotting further demonstrated that full-length dystrophin (Dp427m), absent in M-CMs, was restored in E-CMs (Fig. 2d).

To assess whether this recovery extended to skeletal muscle, the E-iPS cell pair was subjected to a stepwise two-dimensional myogenic differentiation protocol^12^, guiding cells through paraxial mesoderm and myogenic progenitor stages before forming multinucleated myotubes (Fig. 2e, top). The cells progressed through morphological transitions from compact iPS cell colonies (D0) to mesenchymal-like cells (D4–D8), sphere-like aggregates (D16–D24) and finally adherent myoblast-like colonies (D30) (Fig. 2e, bottom). Real-time PCR analysis confirmed the occurrence of stage-specific transcriptional changes, including induction of a presomite marker (*MSGN1*), myogenic progenitor genes (*PAX3, PAX7*) and myoblast/myotube markers (*MYOD1, MYH3, MYOG*) (Fig. 2f) with specific primers (Supplementary Table 1), thereby validating progressive myogenic lineage commitment. The myoblast-like cells were further cultured for an additional 60 days (up to D90), during which they matured into elongated, multinucleated myotubes (Fig. 2g). Notably, myotubes derived from E-iPS cells exhibited robust Dp427m expression, in contrast to myotubes from M-iPS cells, which lacked detectable dystrophin (Fig. 2h, i).

### Patient-derived teratomas as a humanized system for testing ex vivo muscle cell therapy

**Fig. 3:**
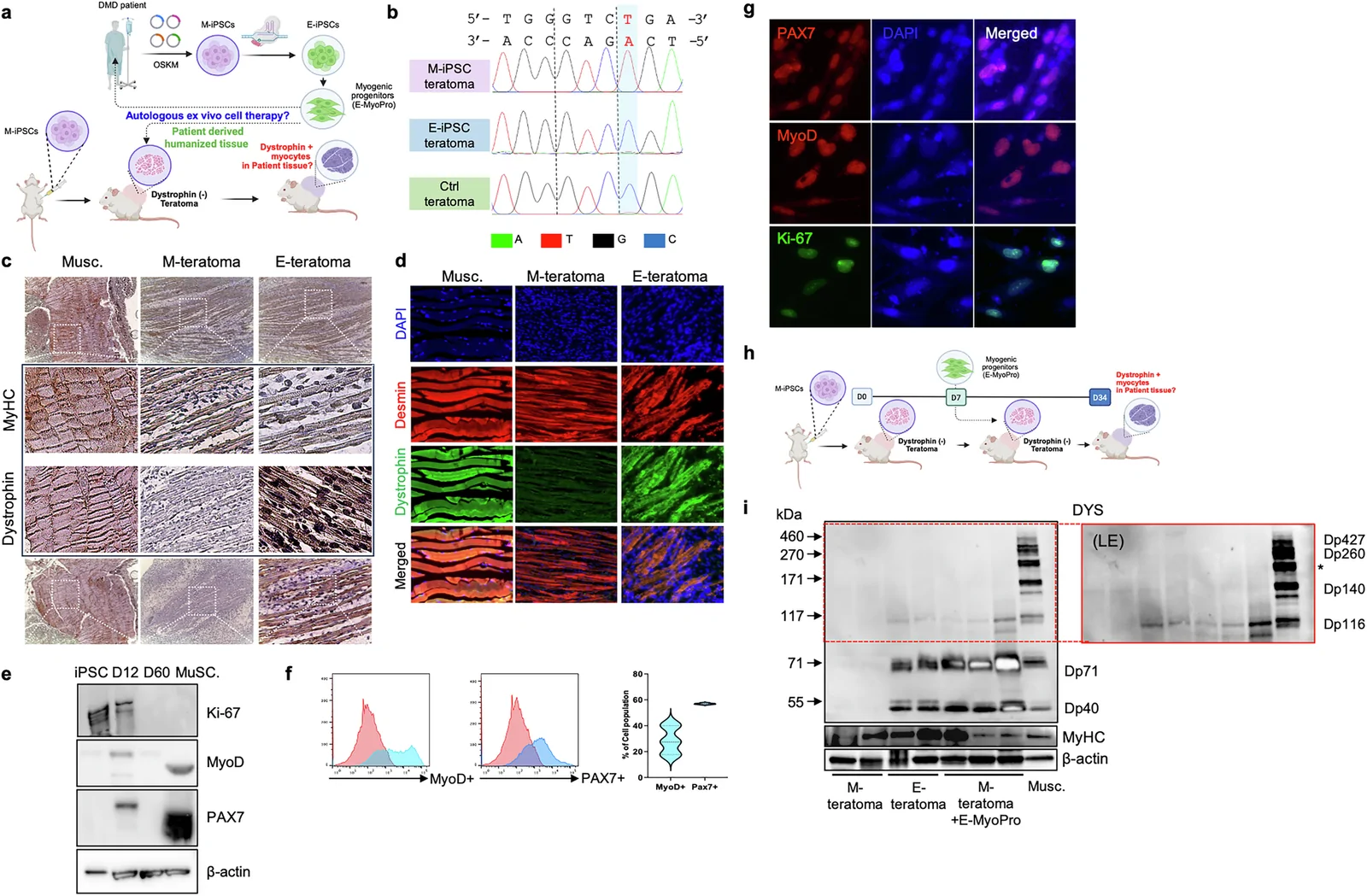
Patient-derived DMD teratoma model for testing ex vivo myogenic progenitor therapy. **a** Experimental schematic illustrating the humanized teratoma model for evaluating autologous E-MyoPro transplantation. **b** Sanger sequencing of M- and E-teratomas verifying the persistence or correction of the exon 6 nonsense mutation. **c** Immunohistochemistry of MyHC and dystrophin in M- and E-teratomas. **d** Immunofluorescence images of desmin (red) and dystrophin (green) in M- and E-teratomas. **e** Immunoblot analysis of Ki-67, MyoD and PAX7 during E-iPS cell myogenic differentiation. **f** Flow cytometry quantification of PAX7^+^ and MyoD^+^ myogenic progenitor populations. **g** Immunofluorescence of Ki-67, PAX7 and MyoD in E-MyoPro. **h** Experimental timeline for transplantation of 1 × 10^5^ E-MyoPro into M-teratomas. **i** Immunoblot of dystrophin isoforms from M-, E- and E-MyoPro-engrafted teratomas, revealing partial restoration of shorter dystrophin isoforms (Dp116, Dp71, Dp40) but absence of full-length Dp427m. Scale bar, 50 μm.

Immunoblotting and immunofluorescence confirmed that iPS cell-derived myogenic progenitors (day 12 of differentiation) matured into myotubes expressing the full-length dystrophin isoform Dp427m (Fig. 2h, i). The recent FDA authorization of a first-in-human trial with off-the-shelf myogenic progenitors (MyoPAXon, NCT06692426) underscores the translational potential of this cell type and motivated us to examine whether gene-corrected myogenic progenitors from E-iPS cells could serve as a source for autologous ex vivo cell therapy in DMD (Fig. 3a). In this regard, a key question to be addressed was whether the demonstrated in vitro differentiation of these progenitors into Dp427m-positive myocytes (Fig. 2h, i) could be replicated in vivo.

Notably, teratomas formed by undifferentiated human pluripotent stem cells in immunodeficient mice recapitulate derivatives of all three germ layers, including skeletal, cardiac and smooth muscle, as well as muscle progenitors, thereby providing a model of multi-lineage human development^35^. We reasoned that teratomas derived from DMD-iPS cells, which lack dystrophin-positive muscle tissue, could serve as a unique patient-specific, humanized platform to assess the efficacy of autologous ex vivo cell therapy with E-iPS cell-derived myogenic progenitors (Fig. 3a). This concept parallels a widely established paradigm in oncology research, where human tumor xenografts in immunodeficient mice are routinely used to evaluate the therapeutic efficacy of drugs or gene-based interventions^36^. By analogy, our DMD-teratoma model adapts this well-validated experimental framework to neuromuscular disease, offering a humanized in vivo system in which to evaluate cell- and gene-based therapeutic strategies.

Before testing this strategy, we validated DMD-iPS cell-derived teratomas (M-teratomas) by confirming retention of the pathogenic mutation (Fig. 3b) and loss of dystrophin expression in MyHC- or desmin-positive muscle-like tissues, whereas robust dystrophin expression was observed in E-iPS cell-derived teratomas (Fig. 3c, d). To model an autologous ex vivo cell therapy setting, we generated myogenic progenitors from E-iPS cells (E-MyoPro, ~day 12 of differentiation), which expressed Ki-67, MyoD and PAX7 as confirmed by immunoblotting (Fig. 3e), flow cytometry (Fig. 3f) and immunofluorescence (Fig. 3g). A total of 1 × 10^5^ E-MyoPro was transplanted into M-teratomas, and the grafts were allowed to engraft for 4 weeks before collection and subsequent analysis of dystrophin restoration (Fig. 3h).

The engraftment of transplanted myogenic progenitors in M-teratomas was evaluated by immunoblotting for dystrophin. As expected, dystrophin signals at ~117, 71 and 55 kDa were completely absent in M-teratomas but readily detected in E-teratomas. Importantly, M-teratomas transplanted with E-MyoPro also showed dystrophin signals comparable to those of E-teratomas. However, neither E-teratomas nor M-teratomas with E-MyoPro exhibited the full-length Dp427m band that was present in mouse muscle (Fig. 3i). These results indicate that gene-corrected myogenic progenitors can engraft and restore expression of multiple dystrophin isoforms—presumably including Dp116, Dp71 and Dp40—within the teratoma environment, although additional lineage maturation or microenvironmental cues may be required to achieve expression of the full-length Dp427m.

### Fabrication of ABE mRNA-loaded LNPs and evaluation of base editing efficiency

**Fig. 4:**
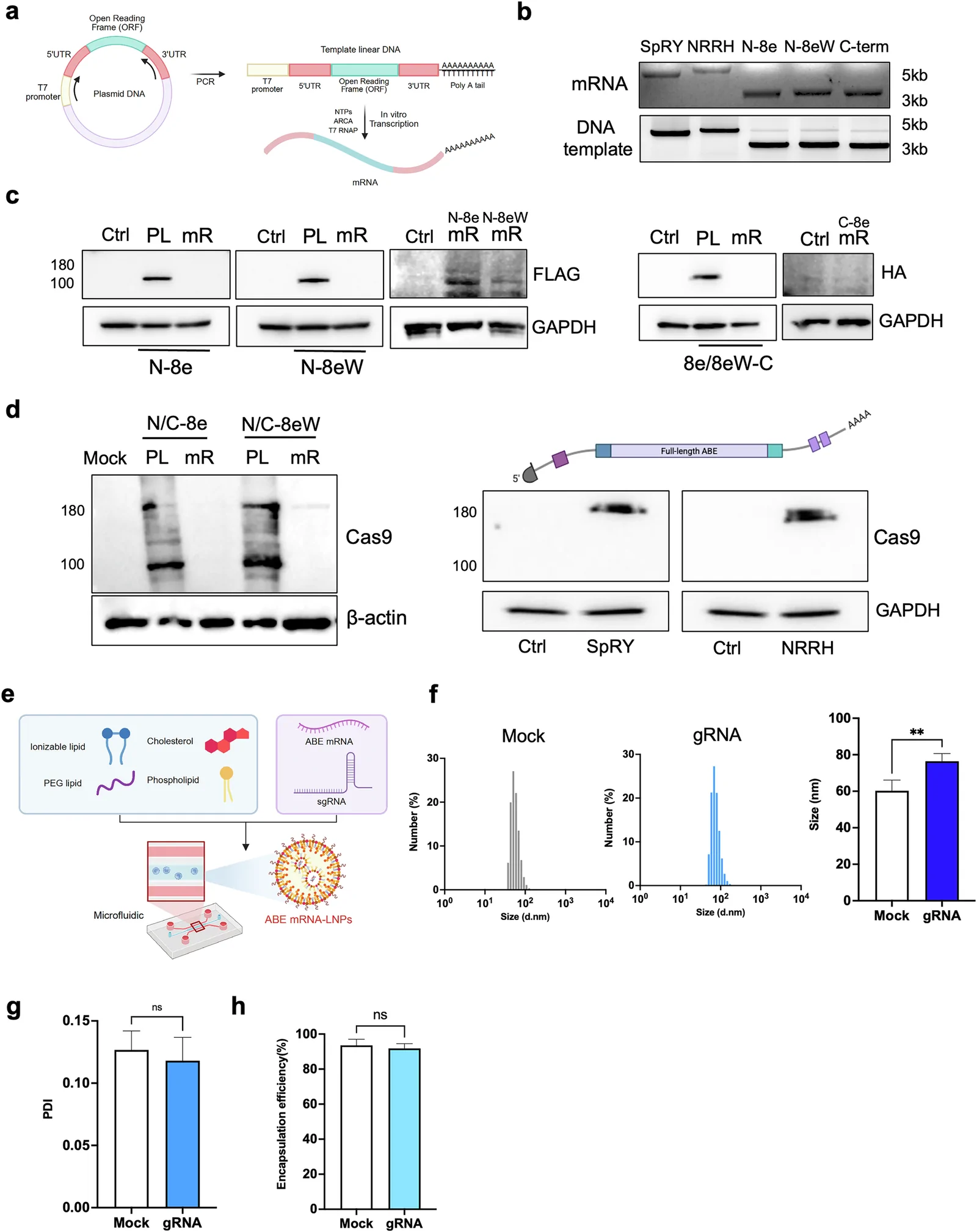
Fabrication of ABE mRNA-loaded LNPs and evaluation of base editing efficiency. **a** Schematic of IVT mRNA synthesis of ABE variants (SpRY, NRRH, 8e, 8eW) including N- and C-terminal split constructs. **b** Gel electrophoresis of in vitro-transcribed mRNAs showing expected transcript sizes compared with plasmid templates. **c** Immunoblot of ABE protein expression (Flag and HA tag) after plasmid or mRNA transfection. **d** Immunoblot showing ABE expression from full SpRY, NRRH and split variants (8e, 8eW) after transfection. **e** Schematic of microfluidic LNP encapsulation process for ABE mRNA and sgRNA. **f** Cryo-TEM image of representative ABE mRNA–LNPs. **g** PDI and encapsulation efficiency of LNPs. **h** DLS analysis revealed comparable particle size distributions for SpRY mRNA–LNPs and SpRY + gRNA mRNA–LNPs, indicating consistent nanoparticle uniformity and stability.

As an alternative to the ex vivo cell therapy approach with E-MyoPro (Fig. 3), we next developed an in vivo gene-editing strategy using LNPs. This nonviral platform was selected to avoid the immunogenicity associated with AAV vectors^37^, which has led to severe—and sometimes fatal—adverse events in DMD clinical settings^38–40^. Because ABE8e is ~5.4 kb—exceeding the ~4.7-kb packaging limit of AAV^41^—mRNA–LNP delivery provides a more suitable modality, as it readily accommodates transcripts of this size. However, long IVT-generated mRNAs are susceptible to premature termination and degradation, resulting in diminished expression and stability^42,43^. To overcome this, we tested whether split ABE mRNAs incorporating Npu intein sequences^44^ at the N- and C-termini could reconstitute full-length functional ABE after translation, as previously demonstrated in AAV systems^41^ (Supplementary Fig. 2a). We therefore synthesized, by IVT, multiple ABE variant mRNAs—including full-length SpRY and NRRH-biased SpRY (NRRH), as well as split forms of ABE8e (8e) and ABE8eW (8eW), which harbor distinct N-terminal fragments (N-8e or N-8eW) but share a common C-terminal fragment (C-term) (Fig. 4a and Supplementary Fig. 2b). Gel electrophoresis confirmed successful generation of both full-length (>5 kb) and split (~3 kb) transcripts (Fig. 4b).

When the N- and C-terminal ABE fragments were delivered as plasmids (PL) or IVT mRNAs (mR) into HEK293T cells, assembly of the full-length Cas9 protein occurred only with plasmid transfection and was completely absent following mRNA transfection (Fig. 4c, d). Consistently, Flag- and hemagglutinin (HA)-tagged split fragments were readily detected from plasmids but not from any split-form mRNA. This discrepancy probably reflects fundamental differences in 5′ untranslated region (5′UTR) architecture between plasmid-driven and IVT-derived expression. Mammalian expression plasmids typically contain a minimal but functional 5′ leader upstream of the start codon and rely on a strong Kozak context for efficient translational initiation. By contrast, when the same sequences are used as T7 IVT templates, the resulting mRNAs often lack a substantive 5′UTR, impairing cap-dependent ribosomal scanning and start-site selection and thereby markedly reducing translation efficiency^45,46^.

This mechanistic limitation explains the negligible protein output from split ABE IVT mRNAs and prompted us to shift our strategy toward full-length ABE constructs. Unexpectedly, full-length ABE plasmids (SpRY and NRRH), which harbor moderately longer 5′ leaders that function as effective UTRs when transcribed in vitro, produced robust Cas9 protein from their IVT mRNAs (Fig. 4d). For in vivo delivery, ABE mRNAs and sgRNAs were encapsulated into LNPs using a microfluidic mixing system. Lipid components—ionizable lipid, 1,2-dioleoyl-*sn*-glycero-3-phosphoethanolamine (DOPE), 1,2-dimyristoyl-*sn*-glycero-3-phosphoethanolamine (DMG)–PEG and cholesterol—were dissolved in ethanol and rapidly combined with an acidic sodium acetate buffer containing ABE mRNA and sgRNA (Fig. 4e). Following formulation, the physicochemical properties of the resulting LNPs were characterized. Cryo-transmission electron microscopy (TEM) revealed uniform spherical particles with a multilamellar internal structure (Supplementary Fig. 2c). Dynamic light scattering (DLS) analysis confirmed a narrow, Gaussian size distribution, with mean diameters of 60.26 ± 5.87 nm for mRNA-only LNPs and 76.38 ± 4.23 nm for mRNA + sgRNA formulations (Fig. 4f), along with a low PDI of 0.1.02 (Fig. 4g). Encapsulation efficiency was further evaluated for LNPs containing ABE mRNA alone and ABE mRNA and sgRNA. This assessment is important because mRNA and synthetic sgRNA differ in their physicochemical properties, and confirming efficient co-encapsulation of both components is critical for effective genome editing. Our results showed that co-encapsulation of mRNA with sgRNA had only a marginal impact on efficiency (Fig. 4h).

### In vivo delivery of ABE mRNA–LNPs restores Dp427m in a humanized DMD teratoma model

**Fig. 5:**
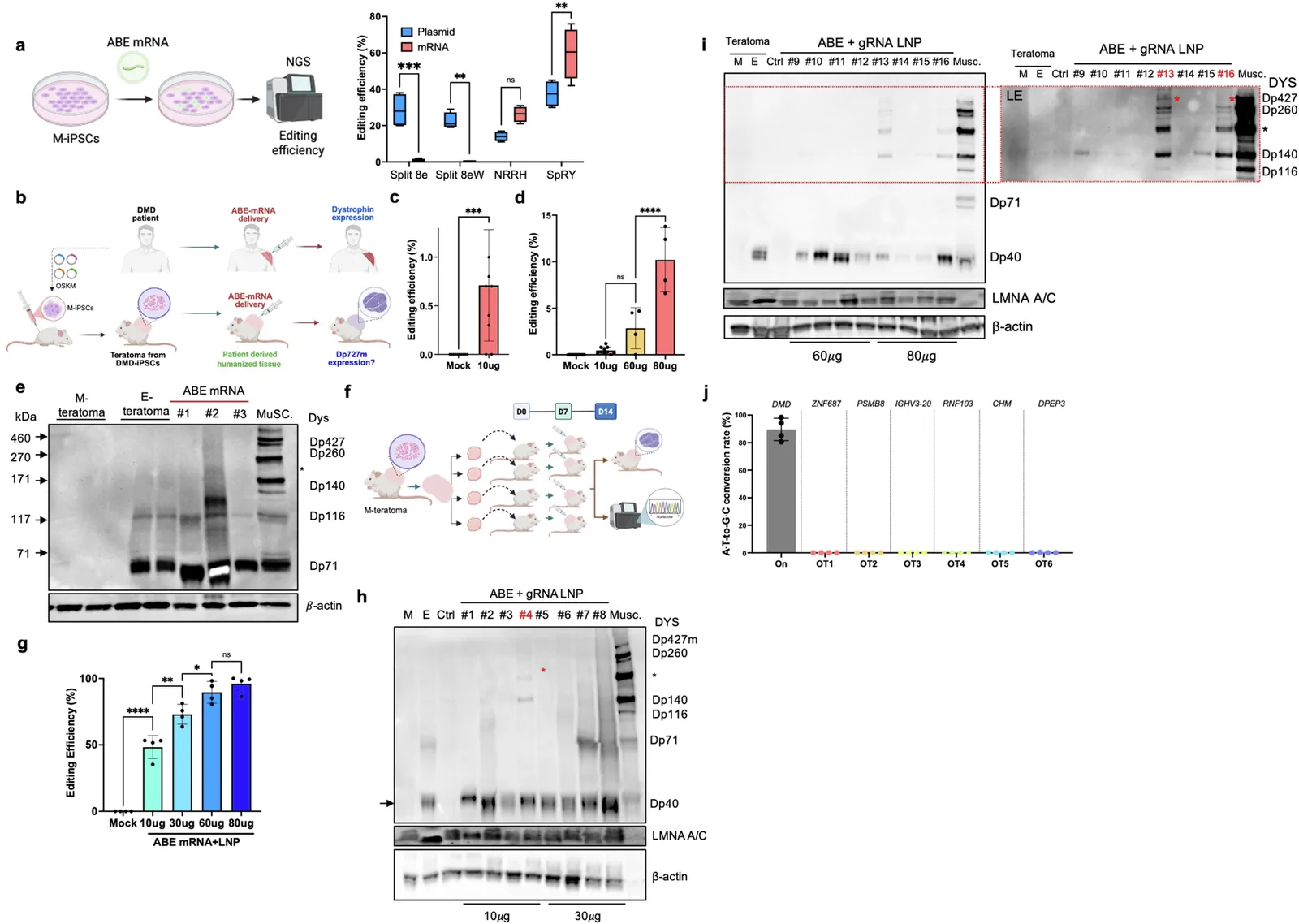
In vivo delivery of ABE mRNA–LNPs restores Dp427m in a humanized DMD teratoma model. **a** Comparative analysis of editing efficiency between plasmid- and mRNA-based delivery for NRRH and SpRY in DMD-iPS cells. **b** Experimental scheme for in vivo injection of ABE mRNA–LNPs into M-teratomas. **c**,**d** Dose-dependent editing efficiency following mRNA–LNP delivery (10–80 μg). **c** Shows the editing result following a single injection of 10 μg SpRY mRNA, whereas **d** shows the dose-dependent editing results obtained with increasing SpRY mRNA doses. **e** Immunoblot showing restoration of isoforms (Dp117, Dp71) in mRNA–LNP-treated group. **f** Secondary transplantation strategy enabling uniform teratoma growth for controlled dose–response analysis. **g** Quantification of editing efficiency by NGS in secondary M-teratomas injected with increasing SpRY mRNA–LNP doses (10–80 μg). **h**,**i** Immunoblot of dystrophin from treated teratomas showing dose-dependent restoration of Dp427m at high-dose (80 μg) delivery, along with partial expression of shorter isoforms (Dp140, Dp116, Dp71, Dp40). **h**,**i** Represent different individual mouse samples: **h** shows samples #1 -8, whereas **i** shows samples #9 -16. **j** Quantification of A-to-G conversion rates at the on-target DMD locus (gray bar) and at predicted off-target exonic sites (OT1 to 6: colored dots) following in vivo delivery of SpRY mRNA–LNPs. Each dot represents an individual off-target site assessed by targeted sequencing. Data represent mean ± s.d.; Student’s *t*-test: *P < 0.05, **P < 0.01, ****P < 0.0001. Scale bar, 50 μm.

Following the successful expression of full-length ABE mRNA conjugated with LNPs (ABE mRNA–LNPs) (Fig. 4), we next delivered ABE mRNA–LNPs together with sgRNA into M-iPS cells and measured editing efficiency (Fig. 5a, left). Strikingly, SpRY mRNA conjugates (SpRY mRNA–LNPs) achieved superior editing outcomes in M-iPS cells, even exceeding the results obtained with plasmid-based delivery (Fig. 5a, right). The successful correction of M-iPS cells with SpRY mRNA–LNPs prompted us to evaluate in vivo editing efficiency in M-teratomas, which recapitulate humanized DMD tissue (Fig. 5b). As an initial test, a single injection of 10 µg SpRY mRNA into M-teratomas (~20 mm in size) yielded ~1% editing efficiency (Fig. 5c). By contrast, escalating the dose to a total of 80 µg SpRY mRNA achieved up to ~15% editing efficiency (Fig. 5d). Protein extracts from M-teratomas adjacent to injection sites of 80 µg SpRY mRNA revealed faint upper dystrophin-associated bands with a smeared appearance (Fig. 5e).

Despite the advantage of recapitulating patient-specific humanized tissue, teratomas are inherently variable, as each develops independently in individual mice, leading to differences in size, composition and maturation that can affect dystrophin restoration. In addition, the heterogeneous differentiation profile—comprising ~20 distinct cell types, including only a small fraction of myogenic progenitors alongside smooth, skeletal and cardiac muscle cells^35^—probably contributes to the isoform variation observed after ABE mRNA delivery. To mitigate these challenges and comply with IACUC guidelines that restrict teratoma size to ~20 mm in the largest dimension, a single primary teratoma was collected at ~20 mm, sectioned into four pieces and transplanted into additional immunodeficient mice (Supplementary Fig. 3a). This secondary transplantation strategy promoted more uniform growth and maturation of teratomas, thereby reducing intersample variability and enabling a more controlled evaluation of ABE mRNA delivery (Fig. 5f). Then, 1 week after transplantation (Supplementary Fig. 3b), varying doses of SpRY mRNA–LNPs were injected, with editing outcomes analyzed another week later (Fig. 5f). To determine whether SpRY mRNA editing could restore full-length Dp427m dystrophin—an outcome not observed with E-MyoPro engraftment (Fig. 3)—we assessed editing efficiency by next-generation sequencing (NGS). Strikingly, SpRY mRNA delivery into these uniformly matured teratomas (~10 mm in length) produced a robust dose-dependent response, achieving editing efficiencies of up to 100% (Fig. 5g and Supplementary Fig. 3c).

Immunoblot analysis further demonstrated dose-dependent restoration of dystrophin isoforms. At 10 µg, all samples exhibited a ~40-kDa dystrophin band (Dp40), with one sample (no. 4) showing additional bands above Dp140 (Fig. 5h). At 60 µg, editing efficiency as indicated by NGS approached near-complete correction, although only partial isoforms were detected at the protein level. Strikingly, in two of four samples treated with 80 µg SpRY mRNA—where editing efficiency closely reached 100%—robust full-length Dp427m was restored, reflected in long-exposure immunoblotting (Fig. 5i). To assess on-target efficiency and potential off-target activity of mRNA–LNP-delivered base editors in human DMD teratomas, we quantified A-to-G conversion at the target DMD locus and at a panel of predicted off-target exonic sites. At the highest dose tested (80 µg), no detectable A-to-G conversion was observed at any of the analyzed six off-target exonic sites, with editing frequencies remaining at background levels comparable to untreated controls (Fig. 5j). Together, these results demonstrate that mRNA–LNP-mediated base editing enables efficient, dose-dependent and highly specific correction of the DMD locus in humanized teratoma tissues without measurable off-target activity, even under high-dose conditions.

### Muscle-enriched teratomas exhibit expression of full-length Dp427m

Interestingly, while full-length Dp427m expression was robustly restored in a subset of teratomas, shorter dystrophin isoforms such as Dp140—normally expressed in brain and fetal tissues—were more frequently detected across multiple SpRY-edited teratomas (Fig. 5i). We hypothesized that this heterogeneity reflects intrinsic variability in teratoma differentiation, whereby even near-complete editing (~100%; Fig. 5g) does not uniformly yield muscle-specific isoform restoration.

**Fig. 6:**
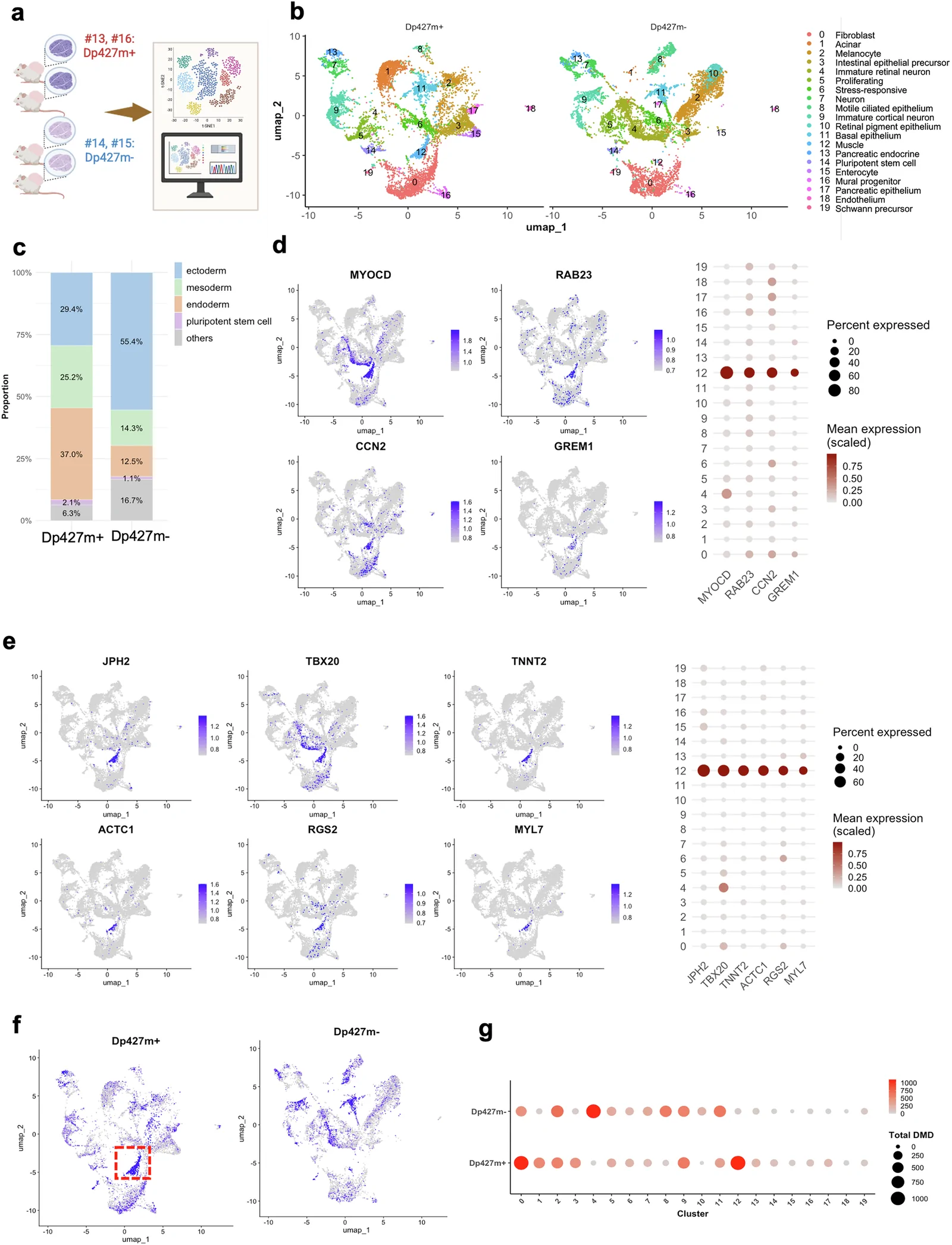
Muscle-enriched teratomas exhibit expression of full-length Dp427m. **a** Schematic overview of the experimental workflow. Dp427m^+^ (no. 13) and Dp427m^−^ (no. 14) teratomas derived from patient iPS cells were subjected to single-nucleus RNA-seq for lineage and muscle-specific expression analysis. **b** UMAP visualization of single-nucleus transcriptomes from Dp427m^+^ and Dp427m^−^ teratomas, clustered into 20 transcriptionally distinct populations. **c** Proportional composition of germ-layer derivatives (ectoderm, mesoderm, endoderm, pluripotent stem cell-like and others) in Dp427m^+^ and Dp427m^−^ teratomas. **d** Feature and dot plots showing enriched expression of canonical skeletal muscle-associated genes (MYOCD, RAB23, CCN2 and GREM1) in cluster 12 of Dp427m^+^ teratomas, indicating enhanced myogenic differentiation. **e** Expression patterns of cardiac muscle markers (JPH2, TBX20, TNNT2, ACTC1, RGS2 and MYL7) demonstrating increased CM maturation in Dp427m^+^ teratomas. **f** UMAP feature plots visualizing DMD expression across both teratoma types. DMD expression was broadly distributed but concentrated within the myogenic cluster (no. 12) in Dp427m^+^ teratomas. **g** Quantitative dot plot showing total DMD transcript distribution across clusters. Dp427m^+^ teratomas contained a markedly higher proportion of DMD-expressing cells within the myogenic cluster (no. 12), consistent with restored Dp427m expression.

To test this, we compared a Dp427m^+^ teratoma (sample 13) and a Dp427m^−^ teratoma (sample 14) using single-cell RNA-seq (Fig. 6a). Guided by previously established teratoma cell-type annotations^35^, we identified 20 subclusters, including muscle, fibroblast and mural progenitor populations (Fig. 6b and Supplementary Fig. 4a). Because teratomas vary in the relative contributions of the three germ-layer derivatives, differences in mesodermal representation naturally influenced the extent of muscle cluster formation (Fig. 6c and Supplementary Fig. 4b). Consistent with this, feature and dot-plot analyses revealed strong enrichment of canonical skeletal muscle markers—*RYR3, LDB3, FBXO32* and *MYOM1*—in cluster 12 of the Dp427m^+^ teratoma, indicative of advanced skeletal muscle differentiation (Fig. 6d). Cardiac muscle clusters in the Dp427m^+^ teratoma likewise showed elevated expression of CM-associated genes (*TNNT2, RYR2, JPH2, LDB3, ACTC1* and *TBX20*), reflecting enhanced cardiac maturation (Fig. 6e).

At the global transcriptomic level, *DMD* expression was broadly distributed across multiple clusters in both Dp427m^+^ and Dp427m^−^ teratomas, reflecting baseline activity of shorter dystrophin isoforms during teratoma differentiation (Fig. 6f). This basal transcription probably explains the consistent detection of shorter isoforms such as Dp40 (~40 kDa) and Dp140 (~140 kDa) in both SpRY-edited and E-teratomas (Fig. 5h, i). Importantly, however, pronounced enrichment of *DMD* expression was localized to the myogenic cluster (no. 12), characterized by skeletal and cardiac muscle markers (Fig. 6d, e), within the Dp427m^+^ teratoma—indicating that the cellular context within this cluster supports translation of full-length Dp427m (Fig. 6g). These differences were clearly visualized in feature plots, where robust *DMD* signal intensity was confined to myogenic lineages of the Dp427m^+^ teratoma (Fig. 6f). Quantitative analysis of cluster composition further confirmed the Dp427m^+^ teratoma to contain a markedly higher proportion of cells within cluster 12 compared with Dp427m^−^, consistent with enhanced commitment toward the myogenic lineage (Supplementary Fig. 4c).

Together, these findings demonstrate that higher teratoma maturation promotes distinct myogenic differentiation and robust expression of Dp427-associated muscle isoforms, underscoring developmental context as a critical determinant of dystrophin restoration efficiency.

## Discussion

Base editing technologies, particularly ABEs, have emerged as powerful in vivo tools that enable precise correction of pathogenic mutations with minimal off-target activity^31,47^. Demonstrating the therapeutic efficacy of these tools requires humanized models carrying patient-specific mutations. However, DMD lacks mutational hot spots and instead exhibits extreme mutational heterogeneity across the *DMD* gene, making the development of variant-specific humanized models technically impractical, particularly for rare mutations.

Here, we establish teratomas derived from patients with DMD as a mutation-inclusive platform in which to evaluate both ex vivo and in vivo therapeutic strategies. Analogous to patient-derived cancer xenografts in oncology^36^, DMD teratomas provide a patient-specific tissue context for therapeutic evaluation. Importantly, restoration of the full-length, muscle-specific dystrophin isoform Dp427m remains the most clinically relevant surrogate for functional recovery^48^, underscoring the translational value of developing a humanized model that can support this readout. In the current teratoma system, however, full-length Dp427m was not reproducibly detected in primary teratomas derived from Edit-iPS cells. Ex vivo transplantation of E-MyoPro into DMD teratomas restored several shorter isoforms but not Dp427m, reflecting the limited myogenic maturation potential of the teratoma environment (Fig. 3h). Similarly, in vivo delivery of SpRY mRNA–LNPs into primary teratomas yielded only faint dystrophin-associated signals without clear restoration of Dp427m (Fig. 5e). To address these limitations, we established secondary teratomas that underwent an additional round of maturation before intervention (Fig. 5f). In this context, SpRY mRNA–LNPs achieved dose-dependent editing efficiencies—approaching 100% in well-matured teratomas—and full-length Dp427m was restored in a subset of high-dose, muscle-enriched samples (Fig. 5i). Notably, shorter isoforms such as Dp116, Dp71 and Dp40 were detected only in teratomas subjected to ex vivo E-MyoPro transplantation (Fig. 3h) or in vivo SpRY mRNA–LNP editing (Fig. 5e, h, i), reflecting the broader cellular contexts in which these isoforms can be expressed during teratoma differentiation. Single-cell RNA-seq analysis showed that the Dp427m-positive secondary teratoma contained an enriched myogenic cluster with elevated DMD expression and skeletal/cardiac muscle-associated markers (Fig. 6e, f). These results support the interpretation that full-length dystrophin recovery in this platform depends not only on efficient mutation correction but also on the presence of sufficiently mature muscle-enriched tissue.

Teratoma growth, size and maturation are inherently difficult to control, and enrichment of mature muscle lineages is restricted by the IACUC-mandated size limit (~20 mm). Thus, although the current platform consistently supports evaluation of mutation correction and shorter dystrophin isoform restoration in a patient-specific human tissue context, reliable assessment of full-length Dp427m will require further optimization to generate matured muscle-enriched teratomas more reproducibly. Future strategies to bias teratoma differentiation toward skeletal muscle, such as inducible myogenic programming, may help generate muscle-enriched teratoma tissues that support more consistent full-length dystrophin expression and enable more reliable local evaluation of patient mutation-specific gene-editing reagents.

In conclusion, patient-derived teratomas represent a scalable and mutation-inclusive humanized system for evaluating gene correction and dystrophin isoform restoration in a patient-specific tissue context. The current study establishes this approach as a proof-of-concept platform for parallel assessment of ex vivo transplantation and in vivo base editing, while also defining myogenic maturation as a key requirement for reproducible full-length Dp427m recovery. Further development of muscle-enriched teratoma models will be required to fully realize the translational potential of this platform for DMD and related monogenic disorders.

## Supplementary information

Supplementary Information

Supplementary Movie 1a

Supplementary Movie 1b

## Data Availability

The targeted deep sequencing data generated in this study have been deposited in the National Center for Biotechnology Information (NCBI) Sequence Read Archive database under accession code BioProject no. PRJNA1476555 https://www.ncbi.nlm.nih.gov/bioproject/PRJNA1476555. The single-cell RNA-seq data generated in this study have been deposited in the Gene Expression Omnibus under accession code GSE334751 https://www.ncbi.nlm.nih.gov/geo/query/acc.cgi?acc=GSE334751. All other data supporting the findings of this study are available from the corresponding author on reasonable request.

## References

[1] Bulaklak, K. & Gersbach, C. A. The once and future gene therapy. *Nat. Commun.* **11**, 5820 (2020). 10.1038/s41467-020-19505-2 PMC7670458 33199717

[2] Chancellor, D., Barrett, D., Nguyen-Jatkoe, L., Millington, S. & Eckhardt, F. The state of cell and gene therapy in 2023. *Mol. Ther.* **31**, 3376–3388 (2023). 10.1016/j.ymthe.2023.11.001 PMC10727993 37927037

[3] Wang, J.-H., Gessler, D. J., Zhan, W., Gallagher, T. L. & Gao, G. Adeno-associated virus as a delivery vector for gene therapy of human diseases. *Signal Transduct. Target. Ther.* **9**, 78 (2024). 10.1038/s41392-024-01780-w PMC10987683 38565561

[4] Ferrari, G., Thrasher, A. J. & Aiuti, A. Gene therapy using haematopoietic stem and progenitor cells. *Nat. Rev. Genet.* **22**, 216–234 (2021). 10.1038/s41576-020-00298-5 33303992

[5] Ebens, C. L. Targeting challenging RDEB skin wounds with gene therapy. *Lancet* **406**, 106–108 (2025). 10.1016/S0140-6736(25)01154-7 40570868

[6] Barrett, D. & Digaudio, D. The landscape of cell and gene therapy today. *Mol. Ther.* **33**, 2316–2323 (2025). 10.1016/j.ymthe.2025.05.006 PMC12172261 40446808

[7] Park, J.-C. et al. Gene editing with ‘pencil’ rather than ‘scissors’ in human pluripotent stem cells. *Stem Cell Res. Ther.* **14**, 164 (2023). 10.1186/s13287-023-03394-5 PMC10283231 37340491

[8] Cerneckis, J., Cai, H. & Shi, Y. Induced pluripotent stem cells (iPSCs): molecular mechanisms of induction and applications. *Signal Transduct. Target. Ther.* **9**, 112 (2024). 10.1038/s41392-024-01809-0 PMC11053163 38670977

[9] Duan, D., Goemans, N., Takeda, S. I., Mercuri, E. & Aartsma-Rus, A. Duchenne muscular dystrophy. *Nat. Rev. Dis. Primers* **7**, 13 (2021). 10.1038/s41572-021-00248-3 PMC10557455 33602943

[10] Biressi, S., Filareto, A. & Rando, T. A. Stem cell therapy for muscular dystrophies. *J. Clin. Invest.* **130**, 5652–5664 (2020). 10.1172/JCI142031 PMC7598050 32946430

[11] Maffioletti, S. M. et al. Efficient derivation and inducible differentiation of expandable skeletal myogenic cells from human ES and patient-specific iPS cells. *Nat. Protoc.* **10**, 941–958 (2015). 10.1038/nprot.2015.057 26042384

[12] Chal, J. et al. Generation of human muscle fibers and satellite-like cells from human pluripotent stem cells in vitro. *Nat. Protoc.* **11**, 1833–1850 (2016). 10.1038/nprot.2016.110 27583644

[13] Chan, S. S.-K. et al. Skeletal muscle stem cells from PSC-derived teratomas have functional regenerative capacity. *Cell Stem Cell* **23**, 74–85 (2018). 10.1016/j.stem.2018.06.010 PMC6752207 29979993

[14] Xie, N., Chu, S. N., Schultz, C. B. & Chan, S. S. Efficient muscle regeneration by human PSC-derived CD82^+^ ERBB3^+^ NGFR^+^ skeletal myogenic progenitors. *Cells* **12**, 362 (2023). 10.3390/cells12030362 PMC9913306 36766703

[15] Azzag, K. et al. Preclinical quality, safety, and efficacy of a CGMP iPSC-derived myogenic progenitor product for the treatment of muscular dystrophies. *Mol. Ther.* **33**, 5099–5117 (2025). 10.1016/j.ymthe.2025.07.007 PMC12338975 40682272

[16] Wang, B., Li, J. & Xiao, X. Adeno-associated virus vector carrying human minidystrophin genes effectively ameliorates muscular dystrophy in mdx mouse model. *Proc. Natl Acad. Sci. USA*. **97**, 13714–13719 (2000). 10.1073/pnas.240335297 PMC17641 11095710

[17] Amoasii, L. et al. Gene editing restores dystrophin expression in a canine model of Duchenne muscular dystrophy. *Science* **362**, 86–91 (2018). 10.1126/science.aau1549 PMC6205228 30166439

[18] Nelson, C. E. et al. In vivo genome editing improves muscle function in a mouse model of Duchenne muscular dystrophy. *Science* **351**, 403–407 (2016). 10.1126/science.aad5143 PMC4883596 26721684

[19] Xu, L. et al. Efficient precise in vivo base editing in adult dystrophic mice. *Nat. Commun.* **12**, 3719 (2021). 10.1038/s41467-021-23996-y PMC8211797 34140489

[20] Lin, J. et al. Adenine base editing-mediated exon skipping restores dystrophin in humanized Duchenne mouse model. *Nat. Commun.* **15**, 5927 (2024). 10.1038/s41467-024-50340-x PMC11251194 39009678

[21] Chemello, F. et al. Precise correction of Duchenne muscular dystrophy exon deletion mutations by base and prime editing. *Sci. Adv.* **7**, eabg4910 (2021). 10.1126/sciadv.abg4910 PMC8087404 33931459

[22] Qiu, H. et al. Efficient exon skipping by base-editor-mediated abrogation of exonic splicing enhancers. *Cell Rep.* **42**, 113340 (2023). 10.1016/j.celrep.2023.113340 37906593

[23] Bulfield, G., Siller, W., Wight, P. & Moore, K. J. X chromosome-linked muscular dystrophy (mdx) in the mouse. *Proc. Natl Acad. Sci. USA*. **81**, 1189–1192 (1984). 10.1073/pnas.81.4.1189 PMC344791 6583703

[24] Yucel, N., Chang, A. C., Day, J. W., Rosenthal, N. & Blau, H. M. Humanizing the mdx mouse model of DMD: the long and the short of it. *npj Regen. Med.* **3**, 4 (2018). 10.1038/s41536-018-0045-4 PMC5816599 29479480

[25] Zhang, Y. et al. A humanized knockin mouse model of Duchenne muscular dystrophy and its correction by CRISPR–Cas9 therapeutic gene editing. *Mol. Ther. Nucleic Acids* **29**, 525–537 (2022). 10.1016/j.omtn.2022.07.024 PMC9398917 36035749

[26] Durbacz, M. Z. et al. Optimized genomic editing of a common Duchenne muscular dystrophy mutation in patient-derived muscle cells and a new humanized mouse model. *Mol. Ther. Nucleic Acids* **36**, 102569 (2025). 10.1016/j.omtn.2025.102569 PMC12167475 40520365

[27] Pickar-Oliver, A. et al. Full-length dystrophin restoration via targeted exon integration by AAV-CRISPR in a humanized mouse model of Duchenne muscular dystrophy. *Mol. Ther.* **29**, 3243–3257 (2021). 10.1016/j.ymthe.2021.09.003 PMC8571168 34509668

[28] Park, J.-C., Kim, K.-T., Jang, H.-K. & Cha, H.-J. Transition substitution of desired bases in human pluripotent stem cells with base editors: a step-by-step guide. *Int. J. Stem Cells* **16**, 234–243 (2023). 10.15283/ijsc22171 PMC10226858 36823978

[29] Park, J.-C. et al. Enhancing genome editing in hPSCs through dual inhibition of DNA damage response and repair pathways. *Nat. Commun.* **15**, 4002 (2024). 10.1038/s41467-024-48111-9 PMC11088699 38734692

[30] Neugebauer, M. E. et al. Evolution of an adenine base editor into a small, efficient cytosine base editor with low off-target activity. *Nat. Biotechnol.* **41**, 673–685 (2023). 10.1038/s41587-022-01533-6 PMC10188366 36357719

[31] Porto, E. M., Komor, A. C., Slaymaker, I. M. & Yeo, G. W. Base editing: advances and therapeutic opportunities. *Nat. Rev. Drug Discov.* **19**, 839–859 (2020). 10.1038/s41573-020-0084-6 PMC7721651 33077937

[32] Xiao, Y.-L., Wu, Y. & Tang, W. An adenine base editor variant expands context compatibility. *Nat. Biotechnol.* **42**, 1442–1453 (2024). 10.1038/s41587-023-01994-3 38168987

[33] Nicholson, L. V. et al. Dystrophin in skeletal muscle I. Western blot analysis using a monoclonal antibody. *J. Neurol. Sci.* **94**, 125–136 (1989). 10.1016/0022-510x(89)90223-2 2693617

[34] Anderson, L. V. & Davison, K. Multiplex western blotting system for the analysis of muscular dystrophy proteins. *Am. J. Pathol.* **154**, 1017–1022 (1999). 10.1016/S0002-9440(10)65354-0 PMC1866550 10233840

[35] McDonald, D. et al. Defining the teratoma as a model for multi-lineage human development. *Cell* **183**, 1402–1419 (2020). 10.1016/j.cell.2020.10.018 PMC7704916 33152263

[36] Sausville, E. A. & Burger, A. M. Contributions of human tumor xenografts to anticancer drug development. *Cancer Res.* **66**, 3351–3354 (2006). 10.1158/0008-5472.CAN-05-3627 16585151

[37] Duan, D. Lethal immunotoxicity in high-dose systemic AAV therapy. *Mol. Ther.* **31**, 3123–3126 (2023). 10.1016/j.ymthe.2023.10.015 PMC10638066 37822079

[38] Philippidis, A. After patient death, FDA places hold on Pfizer Duchenne muscular dystrophy gene therapy trial. *Hum. Gene Ther.* **33**, 111–115 (2022). 10.1089/hum.2022.29211.bfs 35696528

[39] Lek, A. et al. Death after high-dose rAAV9 gene therapy in a patient with Duchenne’s muscular dystrophy. *N. Engl. J. Med.* **389**, 1203–1210 (2023). 10.1056/NEJMoa2307798 PMC11288170 37754285

[40] Lek, A. et al. Meeting Report: 2023 Muscular Dystrophy Association Summit on ‘Safety and Challenges in Gene Therapy of Neuromuscular Diseases’. (SAGE, 2024). 10.3233/JND-240002 39121133

[41] Levy, J. M. et al. Cytosine and adenine base editing of the brain, liver, retina, heart and skeletal muscle of mice via adeno-associated viruses. *Nat. Biomed. Eng.* **4**, 97–110 (2020). 10.1038/s41551-019-0501-5 PMC6980783 31937940

[42] Sahin, U., Karikó, K. & Türeci, Ö mRNA-based therapeutics—developing a new class of drugs. *Nat. Rev. Drug Discov.* **13**, 759–780 (2014). 10.1038/nrd4278 25233993

[43] Kowalski, P. S., Rudra, A., Miao, L. & Anderson, D. G. Delivering the messenger: advances in technologies for therapeutic mRNA delivery. *Mol. Ther.* **27**, 710–728 (2019). 10.1016/j.ymthe.2019.02.012 PMC6453548 30846391

[44] Novikova, O., Topilina, N. & Belfort, M. Enigmatic distribution, evolution, and function of inteins. *J. Biol. Chem.* **289**, 14490–14497 (2014). 10.1074/jbc.R114.548255 PMC4031506 24695741

[45] Karollus, A., Avsec, Ž & Gagneur, J. Predicting mean ribosome load for 5′UTR of any length using deep learning. *PLoS Comput. Biol.* **17**, e1008982 (2021). 10.1371/journal.pcbi.1008982 PMC8136849 33970899

[46] Andreev, D. E. et al. Non-AUG translation initiation in mammals. *Genome Biol.* **23**, 111 (2022). 10.1186/s13059-022-02674-2 PMC9082881 35534899

[47] Kingwell, K. Base editors hit the clinic. *Nat. Rev. Drug Discov.* **21**, 545–547 (2022). 10.1038/d41573-022-00124-z 35831515

[48] Bengtsson, N. E. et al. Comparison of dystrophin expression following gene editing and gene replacement in an aged preclinical DMD animal model. *Mol. Ther.* **30**, 2176–2185 (2022). 10.1016/j.ymthe.2022.02.003 PMC9171147 35143959

