# Supplementary material for: Patient-derived teratomas as a humanized platform for dystrophin restoration by ex vivo cell therapy and in vivo base editing
Source: Exp Mol Med. 2026 Oct 1;58(10):3370–81. doi: 10.1038/s12276-026-01854-5 (PMC13737199; doi:10.1038/s12276-026-01854-5)
Supplement: Supplementary file 1 [file 12276_2026_1854_MOESM1_ESM.pdf]

Patient-Derived Teratomas as a Humanized Platform for Dystrophin Restoration by Ex Vivo Cell Therapy and In Vivo Base Editing

Mihn Jeong Park, Hyeon-Bin Kim, Yuna-Hwang, Yun-Jeong Kim, Ju-Chan Park, Ho-Kyun Lim, Yun-Gwi Park, SungHwan Moon, Hyukjin Lee and Hyuk-Jin Cha

Supplementary Materials and Methods

Supplementary Table1. RT-qPCR primer

Gene	Sequence	
POUF51	FWD	CCTGAAGCAGAAGAGGATCACC
	RVS	AAAGCGGCAGATGGTCGTTTGG
SOX2	FWD	GCTACAGCATGATGCAGGACCA
	RVS	TCTGCGAGCTGGTCATGGAGTT
LIN28	FWD	CCAGTGGATGTCTTTGTGCACC
	RVS	GTGACACGGATGGATTCCAGAC
MSGN1	FWD	AGAGGGAGAAGCTCAGGATGAG
	RVS	GTGTCTGGATCTTGGTGAGAGG
PAX3	FWD	GGCTTTCAACCATCTCATTCCCG
	RVS	GTTGAGGTCTGTGAACGGTGCT
PAX7	FWD	GGAGGATGAAGCGGACAAGAAG
	RVS	AGGTCAGGTTCCGACTCCACAT
MYOD1	FWD	CTCCAACTGCTCCGACGGCAT
	RVS	ACAGGCAGTCTAGGCTCGACAC
MYH3	FWD	CTGGAGGATGAATGCTCAGAGC
	RVS	CCCAGAGAGTTCCTCAGTAAGG
MYOG	FWD	AGTGCCATCCAGTACATCGAGC
	RVS	AGGCGCTGTGAGAGCTGCATTC
TNNT2	FWD	AAGAGGCAGACTGAGCGGGAAA
	RVS	AGATGCTCTGCCACAGCTCCTT
ACTA2	FWD	CTATGCCTCTGGACGCACAACT
	RVS	CAGATCCAGACGCATGATGGCA

Supplementary Figure. 1

a

#	Mutation	Exon site	Affected isoforms	Predicted Phenotype	Predicted Dystrophin Size
1	c.4072-2A>C	Exon 30 (splice acceptor)	Dp427, Dp260, Dp140	Severe: muscle, CNS, retina	Likely frameshift or exon skip (~35–40%)
2	c.1950T>A p.Cys650Ter	Exon 17	All isoforms	Severe: systemic	Truncated (~15–20%)
3	c.2314G>T p.Glu772Ter	Exon 19	All isoforms	Severe: systemic	Truncated (~20–25%)
4	c.4171A>T p.Lys1391Ter	Exon 30	Dp427, Dp260, Dp140	Severe: muscle, CNS, retina	Truncated (~35%)
5	c.433C>T p.Arg145Ter	Exon 6	All isoforms	Severe: systemic (very early stop)	Truncated (~5%)
6	Exon 4-13 deletion	Exons 4–13	All isoforms	Severe: systemic	Out-of-frame: loss of function
7	Exon 2 duplication	Exon 2	All isoforms	Severe: systemic (likely frameshift)	Possibly nonfunctional
8	Exon 8-18 deletion	Exons 8–18	All isoforms	Severe: systemic	Out-of-frame: no functional dystrophin
9	Exon 45 deletion	Exon 45	Dp427, Dp260, Dp140	Mild to moderate (possible therapy)	Potential for exon skipping rescue

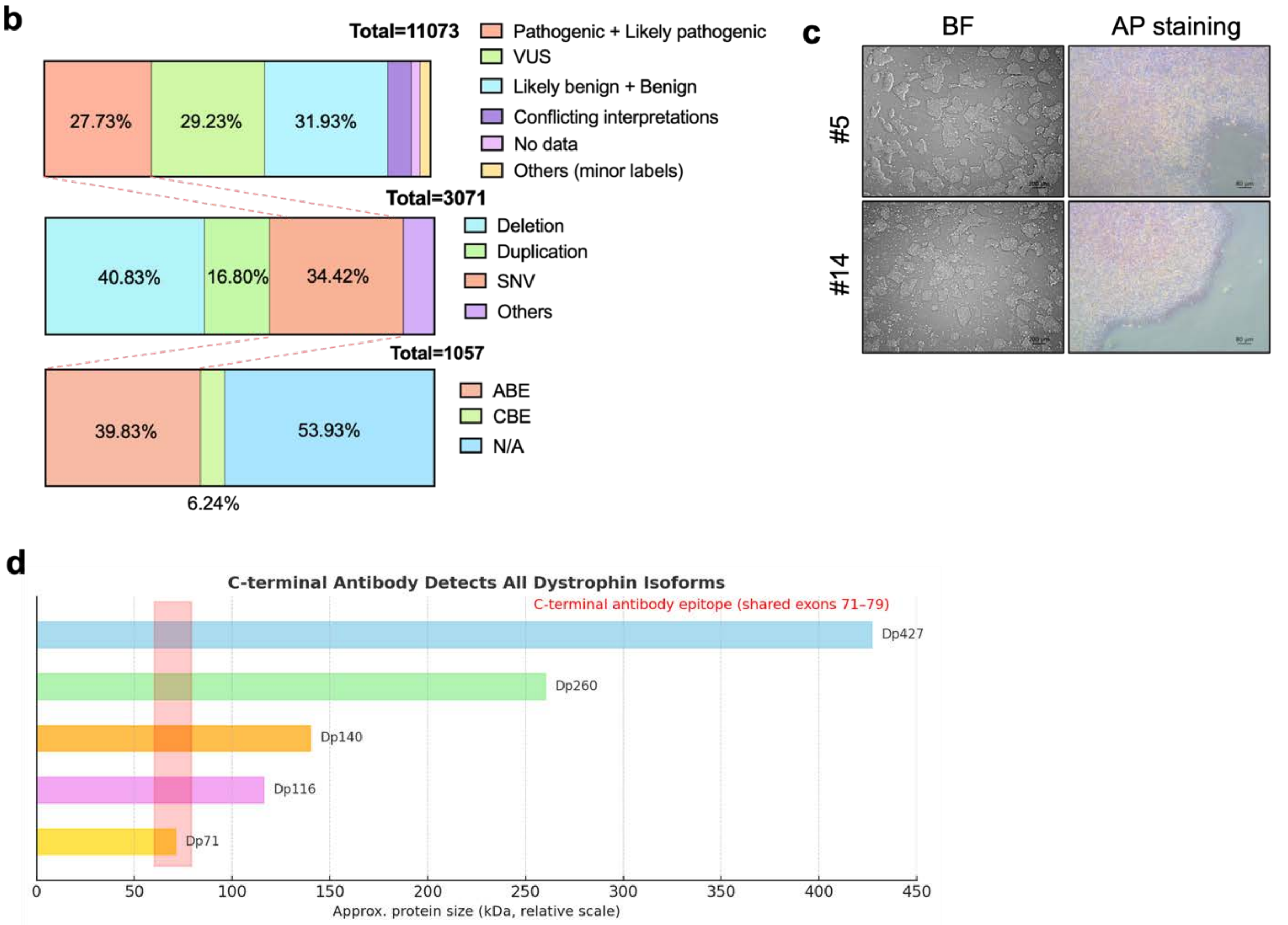

Supplementary Figure 1. (a) Table summarizing nine DMD patient-derived iPSC lines obtained from the Korean National Stem Cell Bank, indicating mutation type, affected exon, isoforms, and predicted clinical severity. (b) Distribution of mutation classes (nonsense, frameshift, splicesite, missense/in-frame) and variant consequences (single-nucleotide, deletion, duplication, indel, insertion) compiled from the ClinVar database. (c) Representative bright-field and alkaline-phosphatase staining images showing typical iPSC morphology and pluripotency of selected lines (#5 and #14). (d) Diagram of dystrophin isoforms recognized by the C-terminal antibody.

Supplementary Figure. 2

a

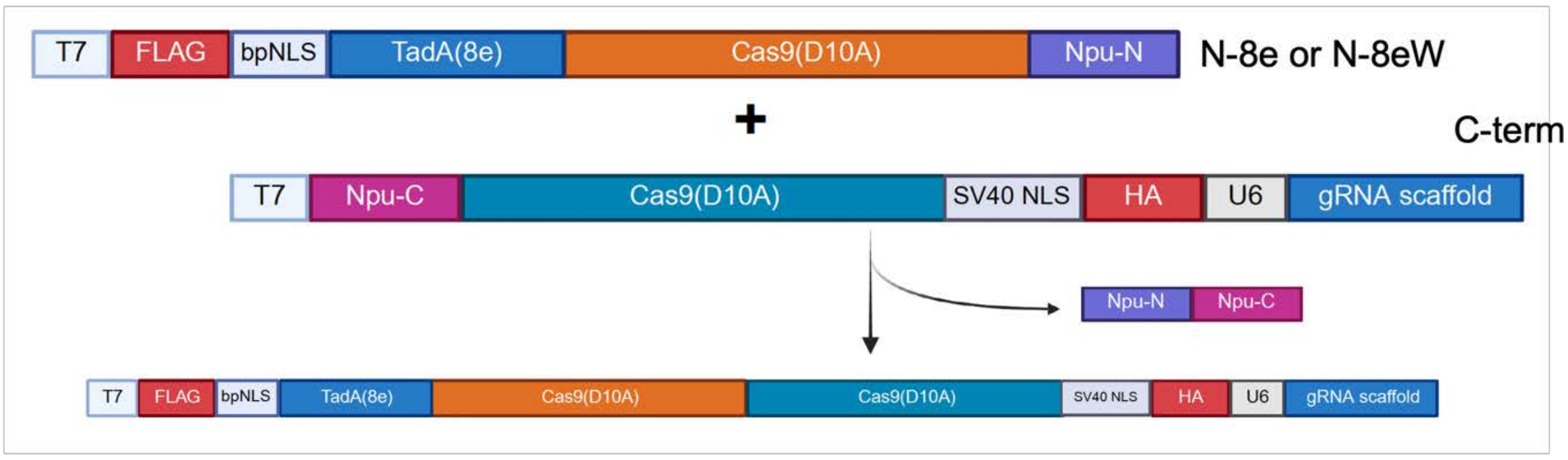

b

ABE	Cas9 backbone	PAM	Split (N and C)	Size	Reference
SpRY	SpRY Cas9	PAM-less	Non-split	4.6kb	#185671
NRRH	SpRY Cas9	NRRH	Non-split		#197502
ABE8e	SpCas9 (D10A)	NGN	N: pAAV-FLAG-NGABEmax8e-NT<br>C: pAAV-HA-NGABEmax-CT	~5.4kb	#197510<br>#197511
ABE8eW	SpCas9 (D10A)	NGN	N: pAAV-FLAG-NGABEmax8eW-NT<br>C: pAAV-HA-NGABEmax-CT	~5.4kb	#197510<br>#197511

c

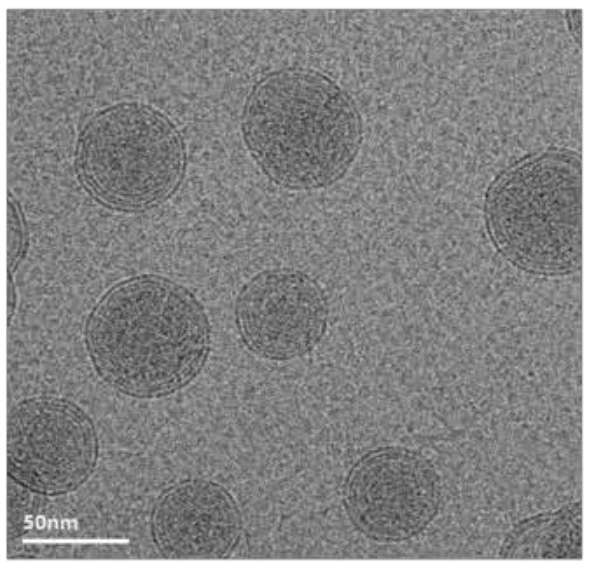

Supplementary Figure 2. (a) Schematic representation of the fusion process of split ABE. Npu-mediated protein splicing between Npu-N and Npu-C reconstitutes the full-length ABE enzyme, enabling targeted adenine base editing. (b) Table summarizing ABE constructs (SpRY, NRRH, ABE8e, ABE8eW) including Cas9 backbone, PAM preference, and split configuration (N/C segments). (c) Cryo-TEM image of representative ABE mRNA–LNPs.

Supplementary Figure. 3

a

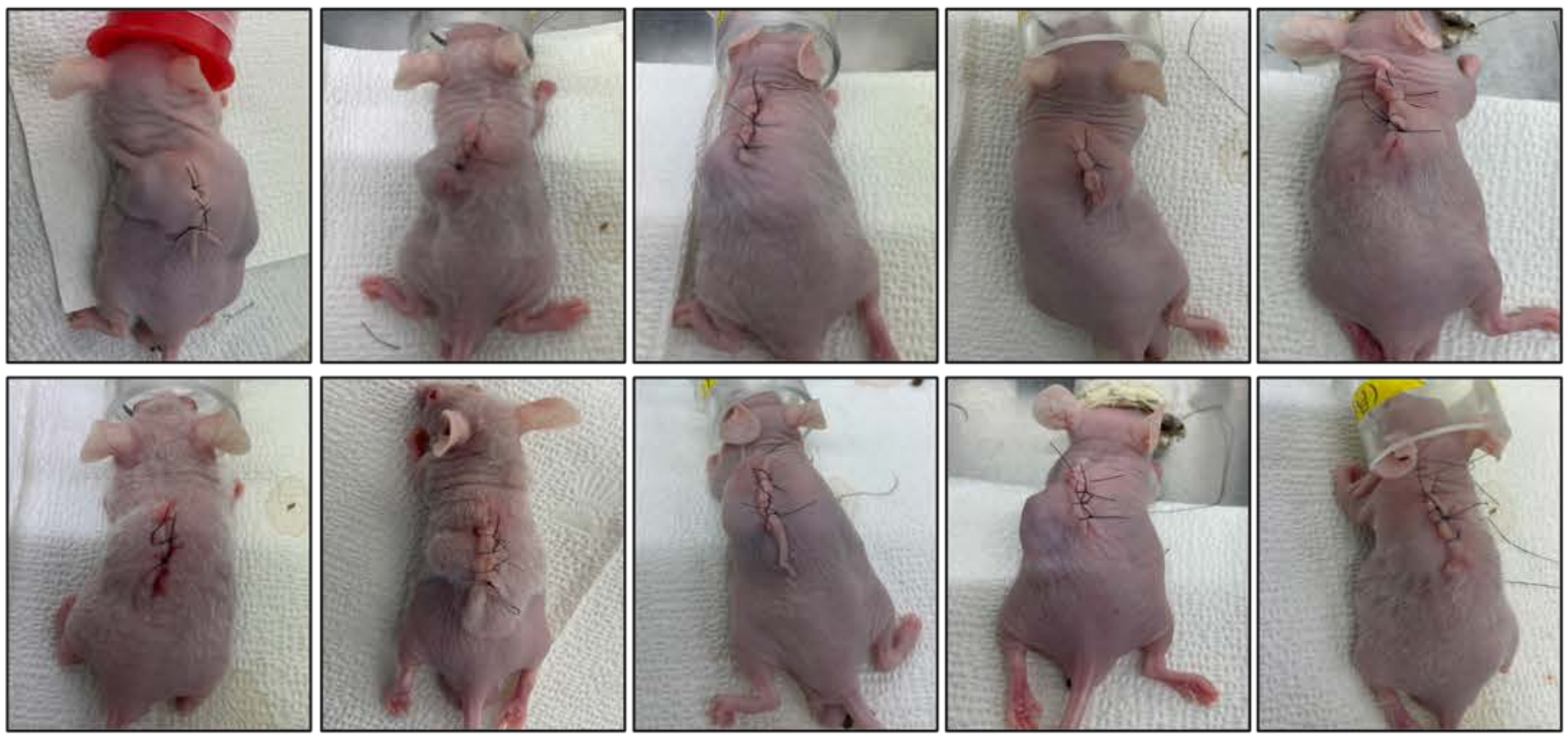

b

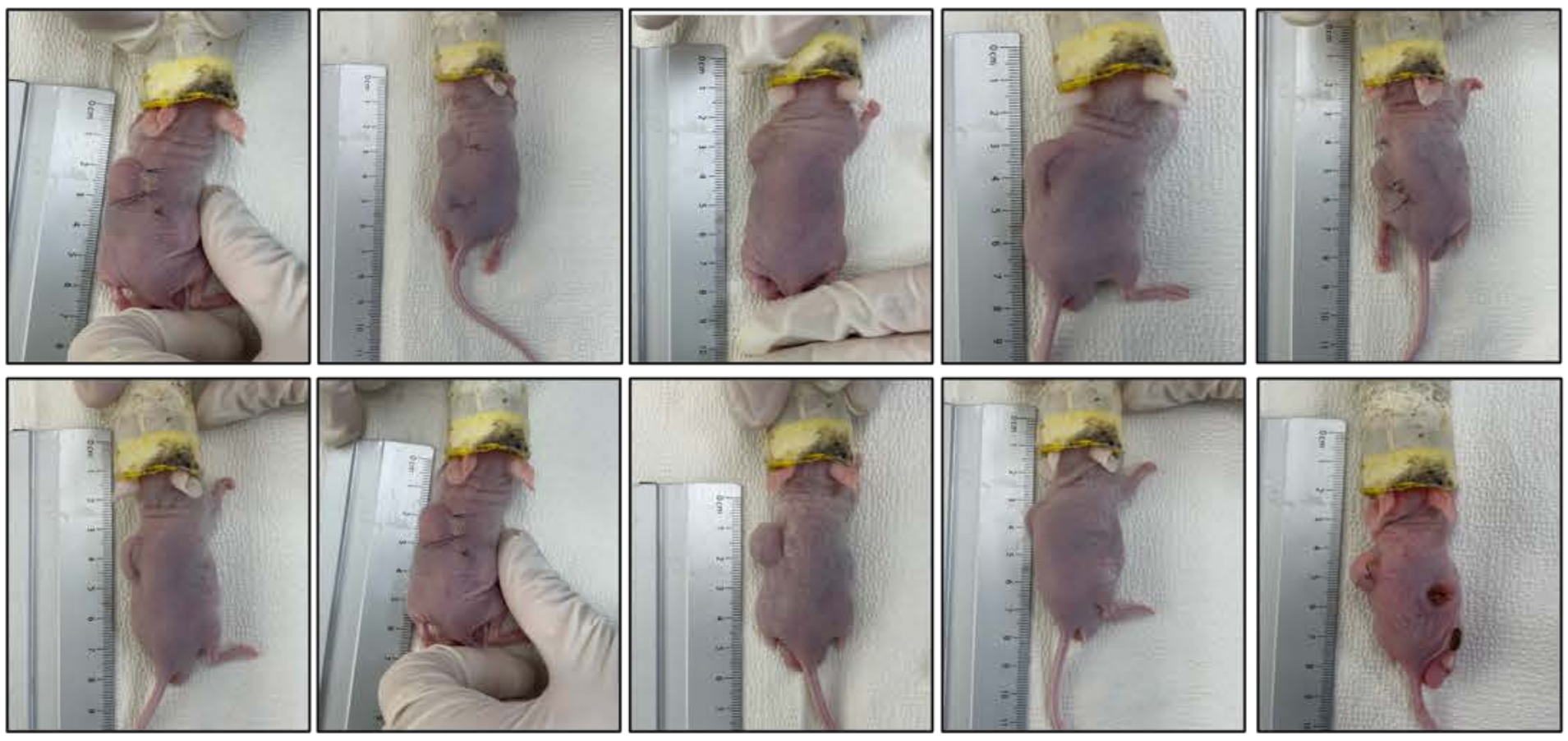

c

Mock

Stop

	C	G	A
A	0.0	0.0	100.0
T	100.0	0.0	0.0
G	0.0	100.0	0.0
C	0.0	0.0	0.0

10 μg

Arg

	C	G	A
A	0.0	0.0	99.9
T	46.8	0.0	0.0
G	0.0	100.0	0.1
C	53.2	0.0	0.0

Stop

	C	G	A
A	0.1	0.0	99.9
T	64.6	0.0	0.0
G	0.0	100.0	0.1
C	35.3	0.0	0.0

Arg

	C	G	A
A	0.0	0.1	99.9
T	48.4	0.0	0.0
G	0.0	99.9	0.1
C	51.6	0.0	0.0

Arg

	C	G	A
A	0.0	0.0	99.9
T	46.7	0.0	0.0
G	0.0	100.0	0.1
C	53.3	0.0	0.0

30 μg

Arg

	C	G	A
A	0.0	0.0	99.9
T	18.6	0.0	0.0
G	0.0	100.0	0.1
C	81.4	0.0	0.0

Arg

	C	G	A
A	0.0	0.0	99.8
T	24.2	0.0	0.0
G	0.0	100.0	0.1
C	75.8	0.0	0.0

Arg

	C	G	A
A	0.0	0.0	99.9
T	28.8	0.0	0.0
G	0.0	100.0	0.1
C	71.2	0.0	0.0

Arg

	C	G	A
A	0.0	0.0	100.0
T	36.2	0.0	0.0
G	0.0	100.0	0.0
C	63.8	0.0	0.0

60 μg

Arg

	C	G	A
A	0.0	0.0	100.0
T	19.3	0.0	0.0
G	0.0	100.0	0.0
C	80.7	0.0	0.0

Arg

	C	G	A
A	0.0	0.0	100.0
T	8.8	0.0	0.0
G	0.0	100.0	0.0
C	91.2	0.0	0.0

Arg

	C	G	A
A	0.0	0.0	100.0
T	14.8	0.0	0.0
G	0.0	100.0	0.0
C	85.1	0.0	0.0

Arg

	C	G	A
A	0.0	0.0	100.0
T	5.7	0.0	0.0
G	0.0	100.0	0.0
C	94.3	0.0	0.0

80 μg

Arg

	C	G	A
A	0.0	0.0	100.0
T	1.7	0.0	0.0
G	0.0	100.0	0.0
C	98.3	0.0	0.0

Arg

	C	G	A
A	0.0	0.0	100.0
T	0.0	0.0	0.0
G	0.0	100.0	0.0
C	100.0	0.0	0.0

Arg

	C	G	A
A	0.0	0.0	100.0
T	0.6	0.0	0.0
G	0.0	100.0	0.0
C	99.4	0.0	0.0

Arg

	C	G	A
A	0.0	0.0	100.0
T	1.8	0.0	0.0
G	0.0	100.0	0.0
C	98.2	0.0	0.0

Supplementary Figure 3. (a) Representative images of mice immediately after secondary teratoma transplantation. (b) Representative images of mice immediately after mRNA-LNP injection one week following secondary teratoma transplantation. (c) Next Generation Sequencing showing progressive editing at the target site with increasing SpRY mRNA dose (10–80 µg).

Supplementary Figure. 4

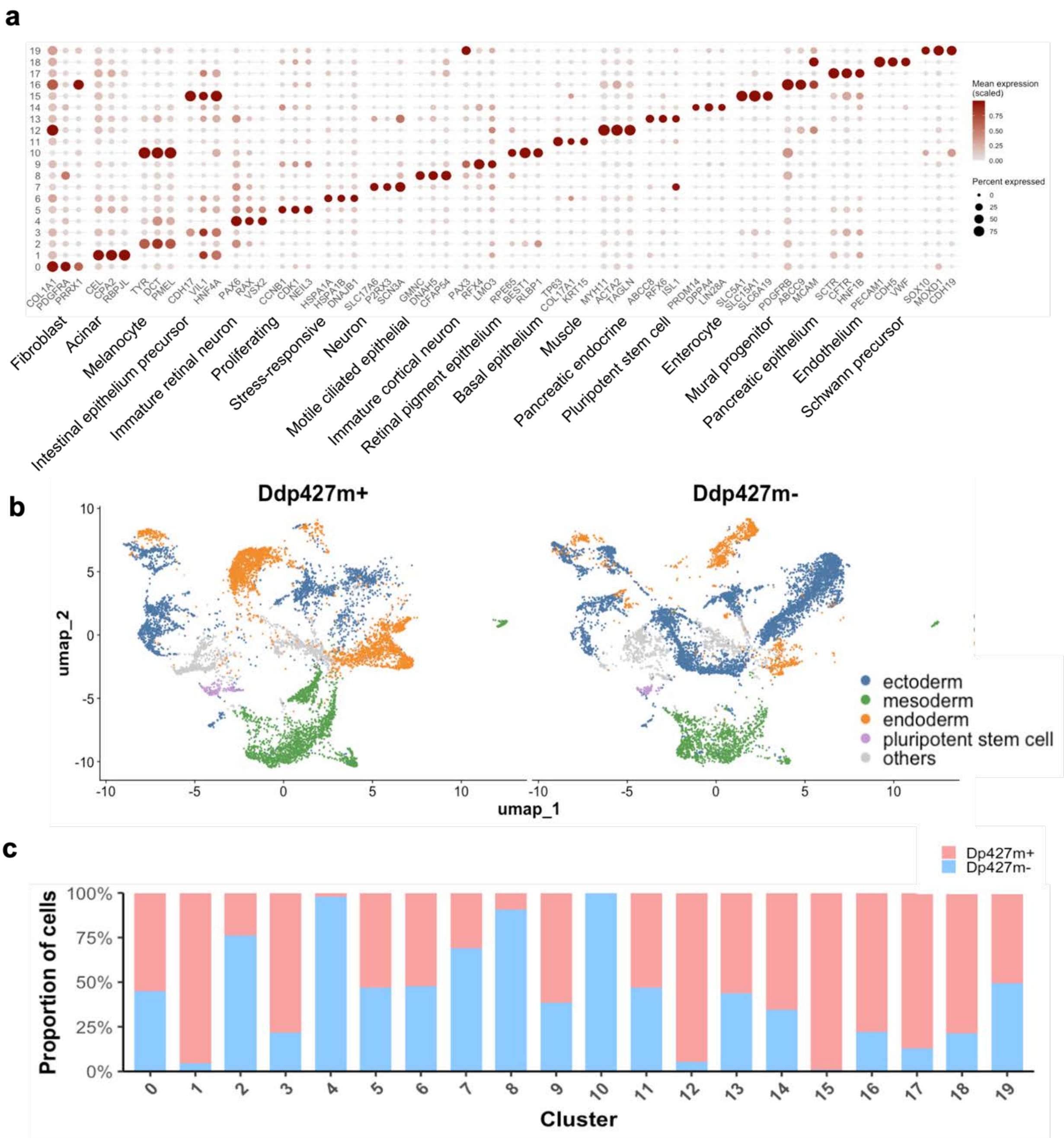

Supplementary Figure 4. (a) Dot plot showing the expression of representative marker genes used for cluster annotation. Each cluster corresponds to a transcriptionally distinct lineage, confirming successful classification of 20 major cell populations. (b) UMAP visualization of Dp427m^{+} and Dp427m^{-} teratomas, color-coded by germ-layer origin (ectoderm, mesoderm, endoderm, pluripotent stem cell–like, and others). (c) Proportion of Dp427m^{+} (red) and

Dp427m^{-} (blue) cells within each cluster, illustrating differences in lineage contribution between the two teratoma groups.

Supplementary Movie 1. Movie 1A M-CM / Movie 1B E-CM
